# Predictive modelling in times of public health emergencies: patients’ non-transport decisions during the COVID-19 pandemic

**DOI:** 10.1186/s12873-025-01340-7

**Published:** 2025-09-11

**Authors:** Hassan Farhat, Cyrine Abid, Guillaume Alinier, Moncef Khadhraoui, Imed Gargouri, Loua Al Shaikh, James Laughton

**Affiliations:** 1 Major Incident Preparedness and Resilience & Ambulance Service Group, Hamad Medical Corporation, PO Box 3050, Doha, Qatar; 2 Laboratory of Epidemiology of Mental Illnesses, Screening and Early Management, Faculty of Medicine “Ibn El Jazzar”, University of Sousse, Sousse, 4000 Tunisia; 3 Laboratory of Screening Cellular and Molecular Processes, Centre of Biotechnology of Sfax, Sfax, Tunisia; 4 University of Hertfordshire, Hatfield, UK; 5 Weill Cornell Medicine-Qatar, Doha, Qatar; 6 Northumbria University, Newcastle upon Tyne, UK; 7 Higher Institute of Biotechnology, University of Sfax, Sfax, Tunisia; 8 Faculty of Medicine, University of Sfax, Sfax, Tunisia

**Keywords:** Pre-hospital, EMS, Non-transport, Machine learning, Prediction

## Abstract

**Background:**

During the COVID-19 pandemic, there was a surge in pre-hospital emergency calls due to the increased prevalence of flu-like symptoms and panic related to the pandemic. However, some patients declined transportation to hospital due to their fear of accessing healthcare facilities. This posed a significant risk to their health outcomes. This study aimed to utilise an extensive dataset, which included the period of the COVID-19 pandemic, in a modern Middle Eastern Emergency Medical Service to comprehend and predict the behaviour of non-transport decisions, a major multi-variable factor in pre-hospital emergency medicine.

**Methods:**

Using Python^®^ programming language, this study employed various supervised machine-learning algorithms, including parametric probabilistic models, such as logistic regression, and non-parametric models, including decision trees, random forest (RF), extra trees, AdaBoost, and k-nearest neighbours (KNN), using a dataset of non-transported patients (refused transport and did not receive treatment versus those who refused transport and received treatment) between 2018 and 2022. Model performance was comprehensively evaluated using Accuracy, F1 score, Matthews correlation coefficient (MCC), receiver operating characteristic area under the curve (ROC AUC), kappa, and R-squared metrics to ensure robust model selection.

**Results:**

From June 2018 to July 2022, 334,392 non-transport cases were recorded. The random forest model demonstrated the best optimised predictive performance, with accuracy = 74.78%, F1 = 0.74, MCC = 0.35, ROC AUC = 0.81, kappa = 0.34, and R-squared = 0.81.

**Conclusion:**

This study indicated that predictive modelling could accurately help identify patients who refuse transport to hospital and may not require treatment on the scene. This enables them to be redirected from the call-taking phase to alternative primary healthcare facilities. This reduces the strain on emergency healthcare resources. The findings suggest that machine learning has the potential to revolutionise pre-hospital care, especially during pandemics, by improving resource allocation and patient outcomes, while highlighting the need for ongoing research to refine these models.

**Supplementary Information:**

The online version contains supplementary material available at 10.1186/s12873-025-01340-7.

## Background

Over the past two decades, the World Health Organisation (WHO) has been working to improve emergency medicine worldwide by implementing various projects in emergency departments [1]. The goal has been to ensure early recognition and management of life-threatening emergencies, thereby contributing to individuals’ well-being and health safety [1]. In line with this, global emergency medical services (EMS) systems have focused on responding to health emergencies in their communities and initiating life-saving treatment in the pre-hospital setting to mitigate and prevent potentially fatal complications. Middle Eastern EMS systems have also evolved rapidly to address the unique challenges in their communities, such as socio-demographic, cultural, and ethnic diversity, which can impact healthcare outcomes [2–4].

Nevertheless, the coronavirus disease 2019 (COVID-19) pandemic has had a significant impact on the global healthcare system, including EMS. The fear of being infected by the virus in hospital settings led to a change in patient behaviour, with many individuals opting not to seek medical attention even in emergencies [5]. This shift in behaviour has highlighted the importance of understanding and predicting patient non-transport decisions in the pre-hospital setting, particularly involving those who refuse transport after receiving treatment and those who refuse transport without any pre-hospital treatment. Non-transport in EMS refers to patients who are evaluated on scene following an emergency call but are not transported to a healthcare facility, regardless of whether they receive treatment on the scene or not [6–8]. In the same context, the Middle East and North Africa (MENA) region has faced various epidemics, including SARS-CoV-2 and MERS, which have further emphasised the need for effective resource allocation and patient management in the pre-hospital setting, considering the Middle Eastern multi-national communities’ particularities and socio-cultural variables. Recent studies in the Ambulance Service in Qatar have revealed that the percentage of patients opting against transportation to emergency departments increased to 40% during the COVID-19 pandemic compared to 23.95% observed during the non-pandemic period [5, 9], which highlights the profound impact of the pandemic on patient behaviour and decision-making processes regarding emergency medical service utilisation.

In such scenarios, artificial intelligence (AI), including machine learning (ML) methods, can predict patient behaviour and optimise resource allocation. By analysing historical data and identifying patterns, AI and ML algorithms can help EMS providers anticipate patient non-transport decisions —those who received treatment and those who did not —and make informed decisions regarding alternative patient care destinations. This approach can help balance preventing hospital overcrowding and ensuring the utilisation of pre-hospital resources only when necessary. This will help ensure more efficient resource utilisation during future pandemics or epidemics.

This study explored the non-transported patient population (who refused transport and did not receive treatment versus those who refused transportation and received treatment) using predictive modelling using ML techniques in a Middle Eastern ambulance service that promotes patient hospital transportation.

## Methods

This was a retrospective quantitative analysis of non-transport patients’ data (refused transport and did not receive treatment versus those who refused transportation and received treatment) provided by the Hamad Medical Corporation Ambulance Service (HMCAS), a modern national Middle Eastern ambulance service at the forefront of delivering pre-hospital emergency care in Qatar [10]. When a medical emergency occurs, individuals can initiate a call for service (CFS) by dialling 999, ensuring rapid emergency medical assistance through call-taking by the emergency medical dispatcher (EMD) and subsequent intervention by paramedics upon ambulance arrival. However, even after receiving the emergency assessment, patients have the right to decline transportation to the hospital, whether or not they received pre-hospital treatment [11, 12]. The prediction horizon for all models in this study was defined as the period from initiating the emergency call to the point of on-scene assessment by paramedics. All predictions were generated using information available up to the crew’s arrival on scene, with the outcome “non-transport decision” determined immediately following this assessment.

The data analysis was performed using Python^®^ programming language. Ethical approval was obtained from the HMC Medical Research Centre (MRC-01-22-264). This article followed the guidelines for Transparent Reporting of a multivariable prediction model for Individual Prognosis or Diagnosis (TRIPOD).

### Source of data

This study involved the analysis of clinical data collected from the HMCAS Business Intelligence registry for the ambulance and hospital electronic patient clinical record (ePCR) system used to create the CFSs [11]. The data was related to all patients who called 999 between June 2018 and July 2022 and were not transported to the hospital. The patients’ identifiers were concealed.

### Population

The study population comprised all patients who contacted HMCAS via 999 for emergency medical assistance in a pre-hospital setting between June 2018 and July 2022, and who, following assessment on scene, were not transported to hospital, irrespective of whether pre-hospital treatment was administered or not. In Qatar, the decision not to transport following pre-hospital emergency assessment is made solely following the patient’s request, to mitigate the risk of misdiagnosis, given that the pre-hospital environment lacks the advanced diagnostic tools and resources available in hospital settings. Non-transported patients were further categorised into three groups:

1. Refused transport and did not receive treatment: Patients who, after assessment by paramedics, declined transport to the hospital and did not receive any form of medical intervention or treatment on scene. This group includes individuals assessed who did not present any immediate clinical need for pre-hospital intervention, or those who explicitly declined treatment and transport. This group of patients may represent lower-acuity cases or those strongly prefer non-engagement with healthcare services.
2. Refused transport but received treatment on scene: Patients who refused transport to the hospital but received medical treatment from the HMCAS crew. The treatment could include administering medications, wound care, or other pre-hospital procedures. These cases might involve patients with serious but manageable conditions who, despite receiving necessary immediate care, chose not to proceed to the hospital for further evaluation. This group of patients often have acute needs addressed on scene, but may remain at risk due to the absence of further hospital-based evaluation.
3. Deceased on arrival (DOA): Emergency calls in which the patient was determined to be deceased upon ambulance arrival at the scene, as per the undeniable death categories defined by the HMCAS clinical practice guidelines where resuscitation efforts are futile, as evidenced by criteria such as rigor mortis, dependent lividity, or injuries incompatible with life [9]. This group reflects operational challenges of the pre-hospital triage system during high-demand periods, such as during the COVID-19 pandemic, when heightened demand and ambulance resources were diverted to non-actionable DOA incidents based on caller-reported cardiac arrests. Such scenarios represented a burden on pre-hospital resources, necessitating their inclusion to assess inefficiencies in emergency response prioritisation during periods of strain. They were considered a public health indicator for health systems during the COVID-19 pandemic [10].

### Data preparation

The dataset comprises 237,862 non-transport cases. Various preprocessing steps were conducted [13, 14]. Data were randomly divided into training (80%, *n* = 190,289) and testing (20%, *n* = 47,573) sets [15]. Continuous response time variables, including CFS creation-to-pending dispatch, pending-to-active dispatch, and creation-to-assigned available, were standardised to a mean of 0 and a standard deviation of 1, using Z-score normalisation, to resolve scale disparities. Categorical variables, such as nationality and chief complaint protocols, were transformed into dummy binary formats [16]. Variables were grouped according to the information presented in Annexe 1. For the identification of outliers, both the Isolation Forest (with a contamination level of 0.01) and the Local Outlier Factor (with novelty set to True) were utilised combined due to their effectiveness in detecting both global outliers (those significantly deviating from the entire dataset) and local outliers (those occurring within the normal range of the dataset) to identify and remove outliers, reducing the training dimensions from 192,089 to 188,38614 [17]. Chi-squared tests were conducted for feature selection to identify and eliminate non-predictive variables [18]. The results of feature selection are presented in Annexe 2. The following variables with the significant p-value (< 0.05) were retained: sex, emergency call zone, dispatched priority, age groups (≤ 14, 14–29, 29–44, 44–59, 59–74, 74–89, ≥ 90), year (2020, 2021) reflecting the pandemic period, month of the year (April, August, January, July, June, March, May, November, October, September), nationality groupings (Middle East and North Africa (MENA), Qatar, South Asia, Sub-Saharan Africa, Europe & Central Asia, North America), chief complaint/Dispatch protocol codes (Abdominal pain, Abnormal behaviour, Allergic reaction, Assault, Back pain, Bleeding, Breathing problem, Cardiac arrest, Chest pain, Choking, Diabetic problem, Drowning/near drowning, Electrocution/lightning, Entrapment, Fall, Fire/burn, HazMat, Headache, Heart problems, Heat-related, Interfacility transport from health centre (Emergency calls originating from primary healthcare centres are considered as community cases due to the non-existence of advanced capabilities at these facilities), Minor illness, Near drowning, Neurological, (Obstetrics and Gynaecology) OBS GYN, Pain, Parental concern, Respiratory, road traffic accidents (RTA), Seizure, Sick person, Toxicology, Traumatic injury, Unconscious, Walking patient), response unit types.

### Outcome

The primary outcome was the non-transport categories, categorised as:

- Refused transport, no treatment (71.24% training, 71.44% testing).
- Refused transport, treated on scene (28.24% training, 27.99% testing).
- DOA (0.51% training, 0.56% testing).

### Predictors

Fifteen variables were retained post-feature selection:

- Demographic: Age, gender, and nationality groupings.
- Clinical: Chief complaints, provisional diagnoses, response unit type and response priority levels.
- Operational: Response time metrics.

### Sample size

From an initial 334,392 cases, 14.0% (*n* = 103,098) with missing values were excluded, identifying 237,862 cases. Post-outlier removal, 188,386 training and 47,519 testing cases were retained.

### Missing data

Missing values (14.0% of the initial dataset) were addressed through listwise deletion, due to the large sample size (*n* = 334,392), retaining 237,862 cases post-deletion, sufficient for robust ML. Further, for example, the variable “Zone_T” (geographic zone name) exhibited 100% missingness across all study years, while “Zone_No” (numeric zone identifier) was fully populated. This pattern arose because HMCAS personnel were protocol-bound to record only one of these mutually exclusive variables. Consequently, “Zone_T”’s missingness is classified as missing not at random, omitted when “Zone_No” was entered, and was excluded to avoid redundancy, ensuring geographic data completeness without introducing bias. Sensitivity analyses comparing retained and excluded cases revealed no significant demographic or clinical differences (age: *p* = 0.32; gender: *p* = 0.45), mitigating concerns about selection bias. Imputation was avoided to prevent introducing spurious associations in time-sensitive predictors. Appendix 3 presents the results of the missing data analysis.

### Statistical analysis: predictive modelling

A supervised ML classification-based method was used for the data analysis to identify patterns and predict values in labelled data [19].

Parametric probabilistic ML algorithms, such as logistic regression (LR), and non-parametric models, including decision trees (DT), random forests (RF), extra trees (ET), AdaBoost, and k-nearest neighbours (KNN), were utilised to develop the predictive model [20]. DTs do not require normalised data and can handle both numerical and categorical variables. Their effectiveness was assessed using the Gini index [20]. ET trains various DTs and aggregates their results [20]. LR is considered an effective classifier for preventing overfitting to the training data, which is particularly useful when dealing with high-dimensional feature spaces [21]. It was assessed using Elastic Net penalty with SAGA solver [20]. KNN, using spatial trees, allows us to identify the variables with the closest distance to the primary variable. RF enables choosing optimised, robust predictions [20]. Adaboost enables a highly accurate, robust classifier by combining multiple poorly performing classifiers. RF and ET benefit from the data with large-scale meta-analyses [20].

Cross-validation, a crucial step to evaluate the model, test its performance, and assess the overfitting, was performed using stratified k-folds (k = 10) based on the weighted average F1 [22]. The number of folds split k = 10 was decided as when the number of folds increased, the variance increased, and the performance indicators’ value was negatively affected. Consequently, the cross-validation decision was made to train the model in the four initial folds and test it in the remaining one.

The models’ performance was measured by calculating the following metrics: “Accuracy”, “Precision, “Recall”, “F1” (the weighted-averaged), “Matthews correlation coefficient (MCC)”, “Receiver Operating Curve-Area Under Curve (ROC AUC)”, “Kappa of Cohen”, and the “coefficient of determination” R^2^ [23, 24] (Table 1).

**Table 1:** Predictive modelling performance metrics

**I) The Confusion Matrix**		
	**Actual positive**	**Actual negative**
Predicted positive	True Positive (TP)	False Positive (FP)
Predicted negative	False Negative (FN)	True Negative (TN)
**II) Non-Transport Variable Confusion Matrix**		
	**Actual positive**	**Actual negative**
	**a) Refused Not Treated**	
Predicted positive	The prediction is positive, and X and Refused were not Treated	The prediction is positive, and X is different from Refused, not Treated
Predicted negative	The prediction is negative, and X refused and was not treated	The prediction is negative, and X is different from Refused, not Treated
**b) Refused and Treated**		
Predicted positive	The prediction is positive, and X Refused and Treated	The prediction is positive, and X is different from Refused and Treated
Predicted negative	The prediction is negative, and X refused and treated	The prediction is negative, and X is different from Refused, not Treated
**III) Metrics Classifiers’ Formulas**		
$\:{A}{c}{c}{u}{r}{a}{c}{y}=\frac{{T}{P}+{T}{N}}{{T}{P}+{T}{N}+{F}{P}+{F}{N}}$	$\:Precision=\frac{TP}{TP+FP}$	
$\:{R}{e}{c}{a}{l}{l}=\frac{{T}{P}}{{T}{P}+{F}{N}}$	$\:F1=\frac{2TP}{2TP+TN+FP}$	
$\:{K}{a}{p}{p}{a}\:\left({K}\right)=\frac{{O}{v}{e}{r}{a}{l}{l}\:{a}{c}{c}{u}{r}{a}{c}{y}-{E}{x}{p}{e}{c}{t}{e}{d}\:{a}{g}{r}{e}{e}{m}{e}{n}{t}}{100-{E}{x}{p}{e}{c}{t}{e}{d}\:{a}{g}{r}{e}{e}{m}{e}{n}{t}}$	$\:ROC\:AUC=\frac{Sensivity}{1-Specifity}$	
$$\:{M}{C}{C}=\frac{{T}{N}\times\:{T}{P}-{F}{N}\times\:{F}{P}}{\surd\:({T}{P}+{F}{P})({T}{P}+{F}{N})({T}{N}+{F}{P})({T}{N}+{F}{N})}$$		

“Accuracy” is the percentage of correct predictions for the test data by comparing the predicted and corrected outputs, improved by modifying the model parameter if needed [25]. It ranges between 0 and 100%; the closer to 100%, the better. The confusion matrix determines this. “Precision” is the proportion of relevant positives among all the examples foreseen to belong to a particular class by determining the amount of relevant data within a sample [25]. “Recall”, also known as “sensitivity”, enables determining the number of selected items within an appropriate sample [25]. The F1 score assesses the accuracy of the testing process [25]. It was utilised to evaluate both precision and recall. It ranges between 1 (perfect precision and recall) and 0 (low precision and recall) [25]. Thence, data can sometimes be biased in the case of a mixture of “Actual Negative” with “Actual Positive” [25]. In this case, F1 and Kappa of Cohen value (K) would better indicate this [25]. Moreover, few studies have identified that the MCC is the best-performing indicator. It considers the true negatives, true positives, false negatives, and false positives and assesses the degree of agreement between actuals and predictions [26, 27]. It ranges between − 1 and 1. The closer to 1, the better. ROC AUC was determined to assess the performance of the binary classifier. It is determined by calculating the True Positive Rate (Sensitivity) divided by the False Positive Rate (Specificity). It ranges between 0 and 1: the closer to 1, the better [28]. R^2^ was also calculated to determine the goodness of a regression model. It ranges between 0 and 1; the closer to 1, the better [29]. Finally, in the case of multi-class classification issues, like this study dataset, Cohen Kappa (k) was also considered to provide robust evidence of the classifier’s performance and reinforce the outcome of the remaining metrics [30]. Table 1 explains the epidemiological benefits of determining these metrics.

Feature importance was determined for the different classifiers. It refers to assessing the input variables and determining which ones are more relevant to the outcome variable [20].

### Risk groups and class imbalance handling

The three predefined risk groups, refused transport with no treatment, refused transport with treatment on scene, and DOA, were retained without further stratification, reflecting Qatar’s pre-hospital non-transport pathways. Given the class imbalance (DOA: 0.5% versus refusal groups: 99.5%), mitigation strategies included stratified ten-fold cross-validation, the use of imbalance-robust performance metrics such as the F1 score and MCC, and the application of the Elastic Net penalty (λ = 0.5) in LR to reduce majority-class bias. RF and ET classifiers were prioritised for their capacity to manage imbalanced data through bootstrap sampling and ensemble voting, with model performance evaluated primarily using the MCC to ensure all confusion matrix elements were considered. Key algorithmic parameters were optimised to enhance clinical relevance: for RF and ET, the number of trees was set to 500, maximum tree depth to 15, and the minimum number of cases required to split a node was adjusted to five; for LR, the strength and balance of regularisation penalties were fine-tuned; for knn, model sensitivity was calibrated by varying the number of neighbours; and for AdaBoost, both the learning rate and the number of estimators were optimised to balance accuracy and robustness. Overfitting was further mitigated by architectural constraints, such as limiting tree depth, and monitoring the training-test accuracy gap, further mitigated overfitting, which was maintained below five per cent. All random processes were seeded (random_state = 42) to guarantee reproducibility. Temporal validation was incorporated by retaining pandemic-era data (2020–2022) in the test set, ensuring that model evaluation reflected the operational challenges encountered during periods of heightened ambulance demand. Although external validation was not performed, the use of stratified data splits (80:20) and the prioritisation of the MCC, which reached 0.81 for the Random Forest model, contributed to the robustness and generalisability of the findings in the context of class imbalance and real-world clinical applicability [31].

## Results

**Fig. 1:**
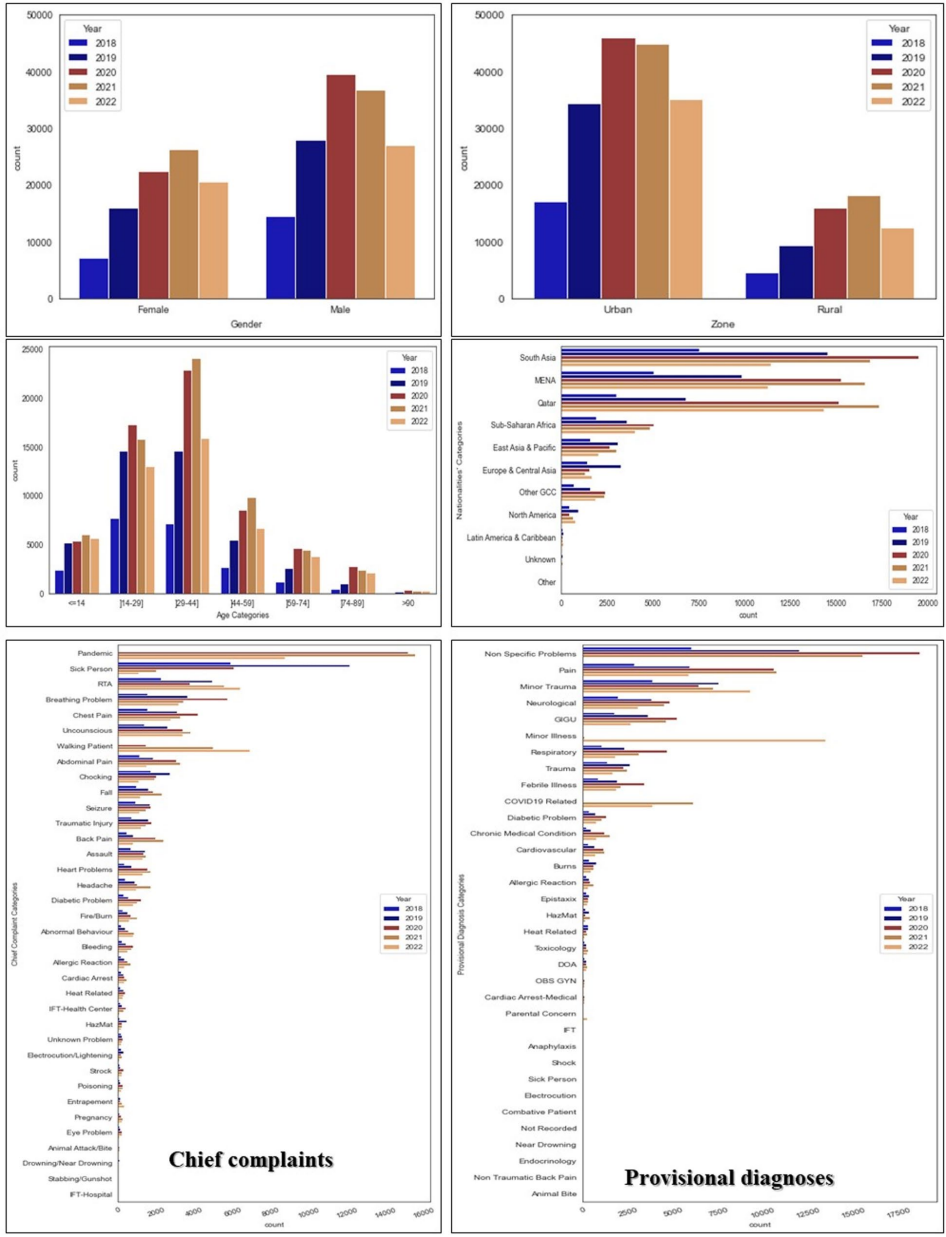
Descriptives information of some of the included variable

From January 2018 to July 2022, HMCAS managed 334,392 non-transport cases. 14.0% of missing values (*n* = 103,0981) were identified and deleted. 237,862 cases of non-transport to the hospital, whether or not they received pre-hospital treatment, were retained after pre-processing, with a mean of 41.74% (*n* = 8,799). Figure 1; Table 2 summarise some of the descriptive information. Non-transport categories were predominantly refusals without treatment (71.24% training, 71.44% testing), followed by refusals with on-scene treatment (28.24% training, 27.99% testing), whilst DOA cases represented 0.51% and 0.56% of the respective datasets. Temporal analysis revealed substantial increases in call volume during 2020–2021, coinciding with the COVID-19 pandemic, particularly affecting urban zones (peak: 47,000 calls in 2020 vs. 35,000 in 2018). Sex distribution remained relatively stable across years, with males comprising approximately 60% of cases. Age-specific patterns showed the highest call volumes amongst middle-aged groups (30–59 years), whilst paediatric cases (≤ 14 years) represented the smallest cohort. Nationality analysis demonstrated that South Asian populations generated the highest call volumes, followed by MENA groups, with Qatari nationals representing a smaller proportion. Chief complaints were dominated by non-specific problems, pain-related presentations, and minor trauma, whilst provisional diagnoses reflected similar patterns, with neurological, obstetric/gynaecological, and minor illness categories featuring prominently.

**Table 2:** Machine learning pre-processing results

**1) «Train»: 80% (*n*** **=** 190,289**) and «Test»: 20% (*n*** **=** **47,573) datasets**						
**2) Standardisation of the continuous variables**						
					**Raw**	**Std**
CFS creation-Pending dispatch (min)				Mean ± std	1.50 ± 4.72	-1.90 × 10^− 16^±1
				[Min – Max]	[0.183–405.23]	[-2.794 × 10^− 01^ − 8.55 × 10^+ 01^]
				25%	0.68	-1.74 × 10^− 01^
				50%	0.88	-1.31 × 10^− 01^
				75%	1.267	-4.99 × 10^− 02^
Pending dispatch-Active dispatch (min)				Mean ± std	0.176 ± 0.3	1.93 × 10^− 01^6±1
				[Min – Max]	[0–48.05]	[-5.87 × 10^+ 01^ − 1.60 × 10^+ 02^]
				25%	0.1	-2.53 × 10^− 01^
				50%	0.13	-1.41 × 10^− 01^
				75%	0.2	8.13 × 10^− 02^
CFS creation-Assigned available (min)				Mean ± std	45.66 ± 106.02	9.44 × 10^− 17^±1
				[Min – Max]	[0.8–5,130.98]	[-4.23 × 10^− 01^ − 4.80 × 10^+ 01^]
				25%	20.6	-2.36 × 10^− 01^
				50%	39.43	-5.88 × 10^− 02^
				75%	55.87	9.62 × 10^− 02^
**3) Shape of the Dataframe after the encoding/transformation**						
	Train			Test		
	Percentage	Dimension		Percentage	Dimension	
		Before	After		Before	After
	80%	(190,29)	-192,09	20%	(47,573)	-47,573
Non-transport Decision	Frequencies (%)			Frequencies (%)		
1 (Refused, not treated)	135,564 (71.24)			33,988 (71.44)		
2 (Refused, treated)	53,746 (28.24)			13,316 (27.99)		
0 (DOA)	979 (0.51)			269 (0.56)		
**4) Outliers detection and splitting**						
Set		Raw Data	Isolation Forest (IF)		Local Outlier Factor	
		Before	After^*^	GridSearch^**^	Basic^a^	GridSearch^b^
TrainSet	X	(192,089)	(188,386)	-190,246	(188,594)	(190,09)
	Y	(192,089.1)	(188,386.1)	(190,246.1)	(188,594.1)	(190,097.1)
TestSet	X	-47,573.12		-47,566.12		-47,519.12
	Y	(47,573.1)		(47,566.1)		(47,519.1)

*Fixed contamination proportion: 0.01

**Contamination: 1$$\:\times\:$$10^−05^

a: Applied on the output of the IF GridSearch with prefixed contamination proportion equal to 0.01

b: Contamination: 1$\:\times\:$10^−05^, Novelty = True

Tables 2 and 3 explain the data pre-processing and feature selection processes. Firstly, the dataset was divided randomly into 80% (*n* = 190,289) for « Train » and 20% (*n* = 47,573) for « Test » datasets.KNN was determined to test the level of the prediction data and the accuracy based on similarity. It selected the subsets with the minor neighbour measures. Secondly, the continuous variable of the duration of the response times (T1: Call record creation to pending dispatch, T2: pending to active dispatch, and T3: creation to assigned available) were standardised. They were rescaled to ensure a balanced contribution. Thirdly, categorical variables were converted into dummy (binary) variables, and the data frame dimensions changed. Fourthly, outlier detection was conducted using the IF and LOF, and the data frame was reshaped. Continuous variables, including call-to-dispatch intervals, were standardised (mean = 0, SD = 1) following outlier removal using IF and LOF algorithms, eliminating 1–3% of observations while preserving dataset integrity. Fifthly, the feature selection was performed using the Chi-squared test. The variables in Table 2 were eliminated (Table 3).

**Table 3:** Variables excluded after feature selection

• Zone: o Urban o Rural		• Chief complaint (Dispatch Protocol code): o Animal Attack Bite. o Bleeding. o Eye Problem. o Pregnancy	
• Years:			
o 2018 o 2019 o 2020	o 2021 o 2022		
• Months:		• Provisional diagnoses:	
o January o February o March o April o May o June	o July o August o September o October o December	o Anaphylaxis. o Chronic Medical Condition. o Diabetic Problem. o Electrocution. o Endocrinology. o Epistaxis.	o Minor Trauma. o Non-specific problems. o Shock o Covid
• Nationality groupings: o East Asia & Pacific o Latin America and the Caribbean o North America o Other GCC o Other			

Table 4 presents the algorithms’ and the metrics’ performances. RF was identified as the best-performing algorithm with 74.78% accuracy, F1 of 74.78%, and MCC and R-squared of 0.81.

**Table 4:** Model performance metrics

	Accuracy Score		F1	MCC	ROC AUC	Kappa (k)	R_squared
	Train	Test					
Decision Tree	99.94%	65.42%	0.667	0.214	0.57	0.213	-0.741
Logistic Regression	73.99%	72.91%	0.692	0.236	0.745	0.212	-0.29
Random Forest	99.94%	74.78%	0.74	0.351	0.811	0.341	0.811
Extra Tree	99.94%	74.39%	0.745	0.366	0.803	0.364	-0.292
Adaboost	73.21%	69.98%	0.65	0.156	0.421	0.134	-0.52
KNN	82.49%	72.88%	0.719	0.303	0.665	0.3	-0.303

**Fig. 2:**
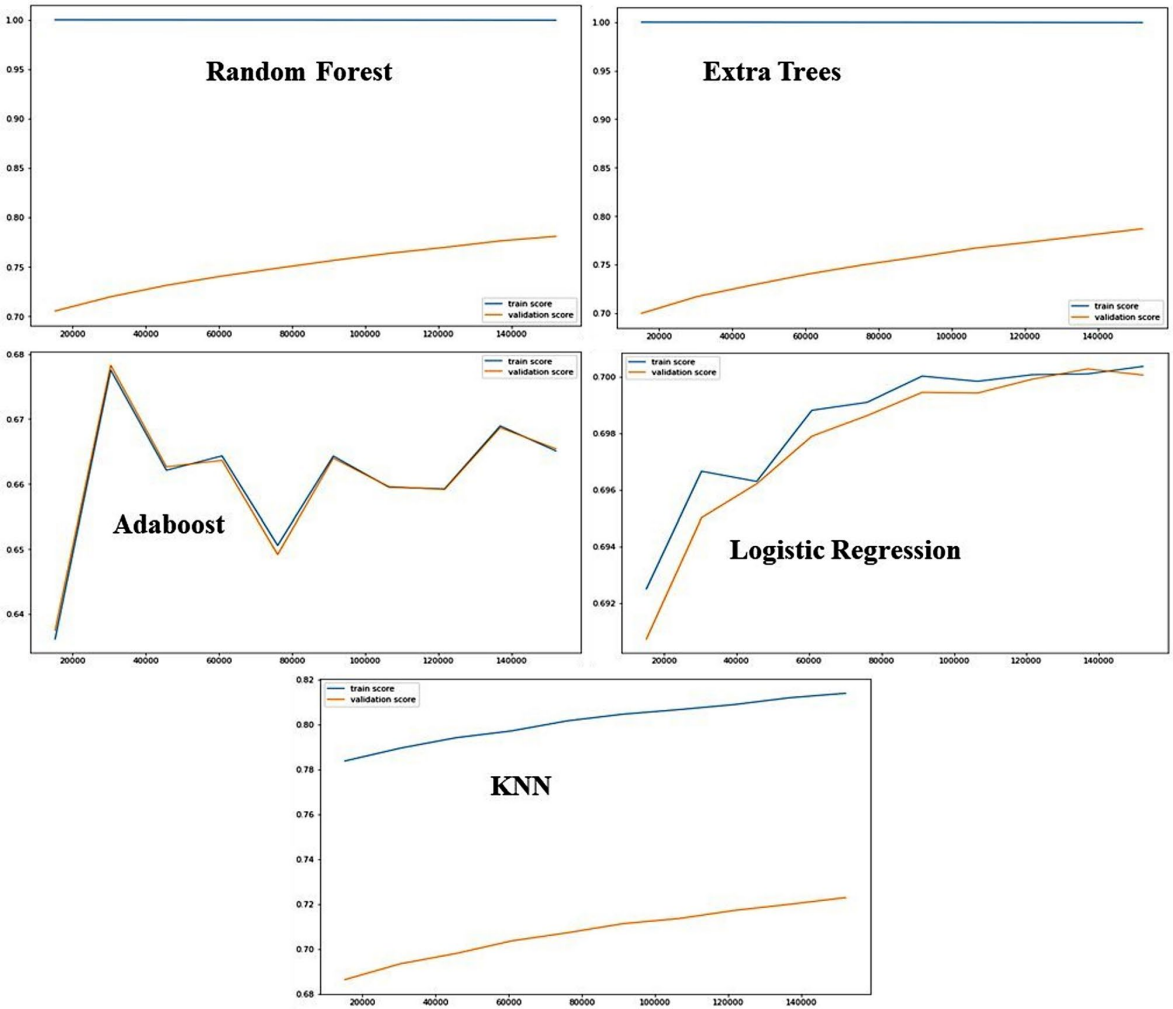
ML algorithms validation performance

The different classifiers were ranked according to feature importance (Fig. 2). The most predictive determining variables for the RF classifier were age groups between 14 and 59, nationalities: Qatari, MENA, and South Asian, “Protocol 1” related to the chief complaint of abdominal pain, Bravo response unit (bike response staffed with bike paramedics with ALS scope of practice), and provisional neurological diagnosis.

## Discussion

The role of ML in healthcare was significantly amplified during the COVID-19 pandemic, particularly in EMS. The COVID-19 pandemic has highlighted the critical need for efficient resource allocation and the importance of predicting healthcare needs to ensure appropriate resource utilisation in healthcare settings [32]. ML’s predictive capabilities can be exploited to anticipate the needs of patients reluctant to seek hospital care due to fear of infection or those who prefer alternative care options [33].

Many patients hesitated to visit hospitals during the pandemic for fear of contracting the virus. This led to a significant shift in the utilisation of pre-hospital emergency healthcare services, with an increase in non-transport decisions by patients. This can be useful for future health emergencies similar to COVID-19. For instance, ML models can identify patients at low risk of severe illness who might opt to decline transport and do not require on-scene emergency treatment. According to insurance policies, they may benefit from being directed to private healthcare centres, which might be less crowded and perceived as safer during a pandemic [34]. These ML models can also predict which patients might be adequately managed through telehealth services, such as those who refused transport and did not receive treatment, thereby reducing the burden on hospital resources and minimising the risk of virus transmission.

As a previous study determined, socio-demographic variables are crucial in determining health outcomes [35]. This study explored the non-transport decisions using a dataset representing more than 200 nationalities. Socio-demographic diversity, such as ethnic and linguistic diversity, can reduce public goods provision, negatively affecting outcomes during public health emergencies [36]. Diverse populations may have different perceptions and attitudes towards seeking healthcare services. These barriers can be especially pronounced during public health emergencies, such as the COVID-19 pandemic, when effective communication and trust in healthcare systems are crucial.

ML has emerged as a powerful tool in public health. Furthermore, in this study, the RF algorithm demonstrated the best overall performance among the models tested, with a test accuracy of 74.80%, an F1 score of 0.74, and an MCC of 0.35. The MCC value provides an overview of the model’s ability to correctly classify all non-transport outcome categories, even in significant class imbalance. Further, an MCC of 0.35 indicates moderate agreement between predicted and actual outcomes, higher than expected by chance, and highlights challenges in reliably predicting rare events such as DOA. The ROC AUC of 0.81 further indicates the model effectively distinguishes the different non-transport groups overall, while a Cohen’s Kappa of 0.34 and R-squared of 0.81 support a moderate level of agreement and explained variance, respectively. Compared to the other algorithms (LR, KNN, Adaboost, and DT), RF outperformed across all metrics, indicating its robustness and suitability for operational decision support in this imbalanced pre-hospital dataset.

Through large and diverse datasets, such as in this study, ML can uncover complex patterns and relationships between socio-demographic factors and non-transport decisions to identify at-risk populations and enable informed targeted interventions. Its ability to handle large, complex datasets makes it particularly suitable for exploring the complex nature of socio-demographic diversity and its impact on public health. It can also provide crucial information about factors influencing non-transport decisions and help develop targeted strategies to improve healthcare access and outcomes for diverse populations, ultimately leading to more effective and equitable healthcare systems. Furthermore, non-specific problems representing low acuity provisional diagnoses have been identified as the predominant category of non-transport patients’ provisional diagnoses during the COVID-19 pandemic. This is robust evidence of the need to revise policies to handle the predicted similar non-transport decisions during public health emergencies by redirecting patients to alternative healthcare facilities instead of dispatching ambulances in critical times like the pandemic and avoiding inefficiencies in EMS unit dispatch. ML has the potential to revolutionise healthcare EMS performance during public health emergencies by identifying low-risk patients who may benefit from being directed to less crowded private healthcare centres or managed through telehealth services, reducing the burden on hospital resources and minimising virus transmission risks. EMS decision-makers can make data-driven decisions to optimise resource allocation, improve healthcare performance and patient outcomes, and reduce the burden on healthcare systems during public health emergencies. In parallel, regular campaigns can help educate the population about the appropriate use of pre-hospital emergency services.

Furthermore, while some studies valued the Deep Intensive Care Unit Central Monitoring System (Deep-ICU CMS), which demonstrates utility in in-hospital cardiac arrest prediction [37], its adoption within Qatar’s pre-hospital EMS would require systemic changes. First, policy revisions would be necessary to transition from the current patient-driven refusal framework to a clinician-guided decision-making model, mandating legal and ethical reforms to grant EMS clinicians authority over transport determinations, a significant departure from Qatar’s autonomy-centric protocols. Concurrent infrastructure upgrades, such as equipping ambulances with wearable sensors for real-time vital sign monitoring and establishing interoperable data pipelines between EMS and hospitals, would be essential to generate the continuous physiological inputs that Deep-ICU CMS relies on. Workflow redesign would necessitate clinician training in interpreting IHCA risk scores during on-scene assessments, directly conflicting with existing patient autonomy norms. Finally, liability management protocols must address potential misalignment between model recommendations and patient refusal rights, particularly in cases where high-risk individuals decline transport despite predictive alerts.

The findings of this study are broadly generalisable to EMS systems across the Gulf Cooperation Council (GCC) countries, which share centralised, government-operated pre-hospital care structures and comparable demographic, cultural, and operational characteristics [38, 39]. Regional analyses confirm that GCC EMS systems uniformly adopt the Anglo-American model, prioritise similar clinical protocols for high-prevalence conditions such as acute trauma and cardio-respiratory emergencies. For instance, studies in Saudi Arabia, Kuwait, and Qatar report nearly identical response time benchmarks (10–15 min), patient refusal rates (~ 30%), and reliance on two-tiered dispatch systems [3, 38]. These shared operational definitions suggest that strategies for managing non-transport categories, such as algorithmic risk stratification and class imbalance mitigation, could be adapted across the region with minimal modification. However, generalisability to non-GCC systems is limited by structural differences. For example, European or North American EMS systems often integrate decentralised, multi-agency models with advanced telemedicine capabilities and stricter adherence to protocol-driven triage, features less prevalent in GCC systems [40].

## Limitations

First, while the RF algorithm demonstrated the highest accuracy and predictive performance in our study, it is essential to acknowledge the limitations associated with this model. One of the primary concerns is the potential for overfitting, as indicated by the discrepancy between the training and testing accuracy scores. The training accuracy reached 99.94%, while the testing accuracy was 74.78%, suggesting that the model may not generalise as effectively to unseen data. This overfitting could lead to overestimating the model’s predictive capabilities in real-world scenarios.

Second, the DOA group’s limited representation was indeed a constraint despite the stratified sampling to mitigate the class imbalance, resulting in the model generalising for DOA prediction. To address this, future research might consider incorporating more advanced feature selection methods to identify and retain only the most relevant predictors that can mitigate overfitting, such as recursive feature elimination, which reduces the risk of overfitting by eliminating redundant or irrelevant features.

Third, excluding cases with missing data, such as incomplete dispatch timestamps, may further bias results, as such exclusions disproportionately affect high-acuity cases where documentation is often prioritised over completeness.

Fourth, the study was retrospective, restricting the ability to capture dynamic changes in future patient behaviours. Restrospective studies introduce risks of selection bias and unmeasured confounding, as data were constrained to pre-existing operational records that lacked granular clinical details such as patient comorbidities and socioeconomic status, or contextual factors, like family preferences, EMS crew experience. These omissions may skew risk predictions, particularly for rare outcomes like DOA.

Fifth, the overfitting risk inherent to tree-based models is evident in the training-test accuracy gap (99.94% vs. 74.78%), likely exacerbated by the inclusion of weakly predictive features such as temporal variables like month/year, which inflated noise.

Prospective studies are needed to validate the model’s predictive power and to ensure that it remains relevant and accurate in the face of evolving healthcare landscapes and patient needs.

## Conclusion

The application of supervised ML algorithms has enabled the development of a highly accurate predictive model during a public health emergency that can identify patients not requiring hospital transport or on-scene treatment during the emergency call-taking phase. This model reduces unnecessary strain on emergency healthcare resources, ensuring availability for those in need by directing suitable patients to alternative healthcare facilities. The study highlights the importance of ML in enhancing decision-making processes within EMS, offering a data-driven approach to effectively managing non-transport decisions.

The research implications extend beyond the COVID-19 pandemic, offering visions for future health emergencies. To translate these predictive models into practice, EMS providers and policymakers should invest in robust digital infrastructure and data integration systems that enable real-time analysis of emergency call data and resource availability. Incorporating ML into contingency plans enables healthcare systems to anticipate better and respond to shifts in patient behaviour and healthcare needs, improving patient outcomes and enhancing healthcare delivery efficiency and effectiveness. The findings demonstrate ML’s potential to revolutionise pre-hospital care during pandemics by ensuring optimal resource allocation. Successful implementation will also require multidisciplinary collaboration, ongoing staff training in using decision-support tools, and the development of clear protocols to ensure that predictive insights are effectively incorporated into operational planning and resource allocation. However, the study acknowledges the limitations of predictive models and the need for ongoing research to refine and validate these tools, focusing on expanding datasets, exploring additional variables, and developing more sophisticated models to enhance the accuracy and applicability of machine learning in pre-hospital care.

## Supplementary Information

Below is the link to the electronic supplementary material.

Supplementary Material 1

## Data Availability

The corresponding author holds the anonymous data supporting this study’s findings. It is available for review upon reasonable request.
