# Supplementary material for: Predictive modelling in times of public health emergencies: patients’ non-transport decisions during the COVID-19 pandemic
Source: BMC Emerg Med. 2025 Sep 11;25:181. doi: 10.1186/s12873-025-01340-7 (PMC12427092; doi:10.1186/s12873-025-01340-7)
Supplement: Supplementary file 1 — Supplementary Material 1 [file 12873_2025_1340_MOESM1_ESM.docx]

# Annexe 1: Variables grouping during data pre-processing

| Nationalities | | | | | |
| --- | --- | --- | --- | --- | --- |
|  | Country | Region |  | Country | Region |
| 1 | American Samoa | East Asia & Pacific | 169 | Algerian | MENA |
| 2 | Australian | East Asia & Pacific | 170 | Algeria | MENA |
| 3 | Australia | East Asia & Pacific | 171 | Djibouti | MENA |
| 4 | Brunei Darussalam | East Asia & Pacific | 172 | Egyptian | MENA |
| 5 | Kingdom of Cambodia | East Asia & Pacific | 173 | Egypt | MENA |
| 6 | Cambodia | East Asia & Pacific | 174 | Iranian | MENA |
| 7 | China | East Asia & Pacific | 175 | Iran | MENA |
| 8 | Fiji | East Asia & Pacific | 176 | Iraqi | MENA |
| 9 | French Polynesia | East Asia & Pacific | 177 | Iraq | MENA |
| 10 | Guam | East Asia & Pacific | 178 | Palestine | MENA |
| 11 | China | East Asia & Pacific | 179 | Palestine State of | MENA |
| 12 | Indonesia | East Asia & Pacific | 180 | Palestinian | MENA |
| 13 | Japan | East Asia & Pacific | 181 | Colonosied territory of Palestine | MENA |
| 14 | Kiribati | East Asia & Pacific | 182 | Jordanian | MENA |
| 15 | Korea | East Asia & Pacific | 183 | Jordan | MENA |
| 16 | Korea South | East Asia & Pacific | 184 | Lebanese | MENA |
| 17 | Korea North | East Asia & Pacific | 185 | Lebanon | MENA |
| 18 | Lao PDR | East Asia & Pacific | 186 | Libya | MENA |
| 19 | Macao SAR, China | East Asia & Pacific | 187 | Morocco | MENA |
| 20 | Malaysia | East Asia & Pacific | 188 | Syrian | MENA |
| 21 | Marshall Islands | East Asia & Pacific | 189 | Syria | MENA |
| 22 | Micronesia, Fed. Sts. | East Asia & Pacific | 190 | Tunisian | MENA |
| 23 | Mongolia | East Asia & Pacific | 191 | Tunisia | MENA |
| 24 | Burma (Myanmar) | East Asia & Pacific | 192 | Western Sahara | MENA |
| 25 | Nauru | East Asia & Pacific | 193 | West Bank and Gaza | MENA |
| 26 | New Caledonia | East Asia & Pacific | 194 | Bermuda | North America |
| 27 | New Zealand | East Asia & Pacific | 195 | Canada | North America |
| 28 | Northern Mariana Islands | East Asia & Pacific | 196 | American | North America |
| 29 | Palau | East Asia & Pacific | 197 | U.S.A | North America |
| 30 | Papua - New Guinea | East Asia & Pacific | 198 | United States Minor Outlying Islands | North America |
| 31 | Hong kong | East Asia & Pacific | 199 | United States | North America |
| 32 | Philippines | East Asia & Pacific | 200 | United Nation | Other |
| 33 | Filipino | East Asia & Pacific | 201 | State of Qatar | Qatar |
| 34 | Samoa | East Asia & Pacific | 202 | Qatari Travel Document | Qatar |
| 35 | Singapore | East Asia & Pacific | 203 | Qatar | Qatar |
| 36 | Solomon Islands | East Asia & Pacific | 204 | Afghanistan | South Asia |
| 37 | Thailand | East Asia & Pacific | 205 | Bangladesh | South Asia |
| 38 | Timor-Leste | East Asia & Pacific | 206 | Bangladeshi | South Asia |
| 39 | Tonga | East Asia & Pacific | 207 | Bhutan | South Asia |
| 40 | Tuvalu | East Asia & Pacific | 208 | Indian | South Asia |
| 41 | Vanuatu | East Asia & Pacific | 209 | India | South Asia |
| 42 | Vietnam | East Asia & Pacific | 210 | Maldives | South Asia |
| 43 | Taiwan (Province of China) | East Asia & Pacific | 211 | Nepalese | South Asia |
| 44 | Tajikistan | East Asia & Pacific | 212 | Nepal | South Asia |
| 45 | Nauru | East Asia & Pacific | 213 | Pakistani | South Asia |
| 46 | Albania | Europe & Central Asia | 214 | Pakistan | South Asia |
| 47 | Andorra | Europe & Central Asia | 215 | Sri Lanka | South Asia |
| 48 | Armenia | Europe & Central Asia | 216 | Angola | Sub-Saharan Africa |
| 49 | Austria | Europe & Central Asia | 217 | Benin | Sub-Saharan Africa |
| 50 | Azerbaijan | Europe & Central Asia | 218 | Botswana | Sub-Saharan Africa |
| 51 | Belarus | Europe & Central Asia | 219 | Burkina Faso | Sub-Saharan Africa |
| 52 | Belgium | Europe & Central Asia | 220 | Burundi | Sub-Saharan Africa |
| 53 | Bosnia and Herzegovina | Europe & Central Asia | 221 | Cabo Verde | Sub-Saharan Africa |
| 54 | Bulgaria | Europe & Central Asia | 222 | Cameroon | Sub-Saharan Africa |
| 55 | Channel Islands | Europe & Central Asia | 223 | Central African Republic | Sub-Saharan Africa |
| 56 | Croatia | Europe & Central Asia | 224 | Republic of Chad | Sub-Saharan Africa |
| 57 | Cyprus | Europe & Central Asia | 225 | Chad | Sub-Saharan Africa |
| 58 | Czech Republic | Europe & Central Asia | 226 | Comoros | Sub-Saharan Africa |
| 59 | Denmark | Europe & Central Asia | 227 | Republic of Congo | Sub-Saharan Africa |
| 60 | Estonia | Europe & Central Asia | 228 | Congo | Sub-Saharan Africa |
| 61 | Faeroe Islands | Europe & Central Asia | 229 | Cote d'Ivoire | Sub-Saharan Africa |
| 62 | Finland | Europe & Central Asia | 230 | Equatorial Guinea | Sub-Saharan Africa |
| 63 | French | Europe & Central Asia | 231 | State of Eritrea | Sub-Saharan Africa |
| 64 | France | Europe & Central Asia | 232 | Eritrea | Sub-Saharan Africa |
| 65 | Georgia Republic | Europe & Central Asia | 233 | Eswatini | Sub-Saharan Africa |
| 66 | Georgia | Europe & Central Asia | 234 | Federal Democratic Republic of Ethiopia | Sub-Saharan Africa |
| 67 | Federal Republic of Germany | Europe & Central Asia | 235 | Ethiopia | Sub-Saharan Africa |
| 68 | Germany | Europe & Central Asia | 236 | Gabon | Sub-Saharan Africa |
| 69 | Gibraltar | Europe & Central Asia | 237 | Gambia, The | Sub-Saharan Africa |
| 70 | Greece | Europe & Central Asia | 238 | Ghana | Sub-Saharan Africa |
| 71 | Greenland | Europe & Central Asia | 239 | Republic of Guinea | Sub-Saharan Africa |
| 72 | Hungary | Europe & Central Asia | 240 | Guinea | Sub-Saharan Africa |
| 73 | Iceland | Europe & Central Asia | 241 | Guinea-Bissau | Sub-Saharan Africa |
| 74 | Lexumbourg | Europe & Central Asia | 242 | Kenya | Sub-Saharan Africa |
| 75 | Irish | Europe & Central Asia | 243 | Lesotho | Sub-Saharan Africa |
| 76 | Ireland | Europe & Central Asia | 244 | Liberia | Sub-Saharan Africa |
| 77 | Isle of Man | Europe & Central Asia | 245 | Madagascar | Sub-Saharan Africa |
| 78 | Italy | Europe & Central Asia | 246 | Malawi | Sub-Saharan Africa |
| 79 | Kazakhstan | Europe & Central Asia | 247 | Mali | Sub-Saharan Africa |
| 80 | Kosovo | Europe & Central Asia | 248 | Mauritania | Sub-Saharan Africa |
| 81 | Kyrgyz Republic | Europe & Central Asia | 249 | Mauritius | Sub-Saharan Africa |
| 82 | Latvia | Europe & Central Asia | 250 | Mozambique | Sub-Saharan Africa |
| 83 | Liechtenstein | Europe & Central Asia | 251 | Namibia | Sub-Saharan Africa |
| 84 | Lithuania | Europe & Central Asia | 252 | Niger | Sub-Saharan Africa |
| 85 | Luxembourg | Europe & Central Asia | 253 | Nigeria | Sub-Saharan Africa |
| 86 | Moldova | Europe & Central Asia | 254 | Rwanda | Sub-Saharan Africa |
| 87 | Monaco | Europe & Central Asia | 255 | Sao Tome and Principe | Sub-Saharan Africa |
| 88 | Montenegro | Europe & Central Asia | 256 | Senegal | Sub-Saharan Africa |
| 89 | Netherlands (Holland) | Europe & Central Asia | 257 | Seychelles | Sub-Saharan Africa |
| 90 | Netherlands | Europe & Central Asia | 258 | Sierra Leone | Sub-Saharan Africa |
| 91 | North Macedonia | Europe & Central Asia | 259 | Federal Republic of Somalia | Sub-Saharan Africa |
| 92 | Norway | Europe & Central Asia | 260 | Somalia | Sub-Saharan Africa |
| 93 | Polish | Europe & Central Asia | 261 | South Africa | Sub-Saharan Africa |
| 94 | Poland | Europe & Central Asia | 262 | Sudan | Sub-Saharan Africa |
| 95 | Portugal | Europe & Central Asia | 263 | Sudanese | Sub-Saharan Africa |
| 96 | Romania | Europe & Central Asia | 264 | Republic of the Sudan | Sub-Saharan Africa |
| 97 | Russian Federation | Europe & Central Asia | 265 | South Sudan | Sub-Saharan Africa |
| 98 | San Marino | Europe & Central Asia | 266 | Sudan | Sub-Saharan Africa |
| 99 | Serbia | Europe & Central Asia | 267 | Tanzania | Sub-Saharan Africa |
| 100 | Slovak Republic | Europe & Central Asia | 268 | Togo | Sub-Saharan Africa |
| 101 | Slovenia | Europe & Central Asia | 269 | Uganda | Sub-Saharan Africa |
| 102 | Spain | Europe & Central Asia | 270 | Zambia | Sub-Saharan Africa |
| 103 | Sweden | Europe & Central Asia | 271 | Zimbabwe | Sub-Saharan Africa |
| 104 | Switzerland | Europe & Central Asia | 272 | Unknown | Unknown |
| 105 | Tajikistan | Europe & Central Asia |  |  |  |
| 106 | Turkey | Europe & Central Asia |  |  |  |
| 107 | Turkmenistan | Europe & Central Asia |  |  |  |
| 108 | Ukraine | Europe & Central Asia |  |  |  |
| 109 | British | Europe & Central Asia |  |  |  |
| 110 | British Indian Ocean Island | Europe & Central Asia |  |  |  |
| 111 | United Kingdom | Europe & Central Asia |  |  |  |
| 112 | Uzbekistan | Europe & Central Asia |  |  |  |
| 113 | Malta | Europe & Central Asia |  |  |  |
| 114 | Bosnia | Europe & Central Asia |  |  |  |
| 115 | Hungarian | Europe & Central Asia |  |  |  |
| 116 | Bahrain | GCC Other |  |  |  |
| 117 | Kuwait | GCC Other |  |  |  |
| 118 | Oman | GCC Other |  |  |  |
| 119 | Saudi Arabia | GCC Other |  |  |  |
| 120 | United Arab Emirates | GCC Other |  |  |  |
| 121 | Yemen | GCC Other |  |  |  |
| 122 | Yemeni | GCC Other |  |  |  |
| 123 | Republic of Yemen | GCC Other |  |  |  |
| 124 | Antigua and Barbuda | Latin America & Caribbean | 167 | Venezuela, RB | Latin America & Caribbean |
| 125 | Argentina | Latin America & Caribbean | 168 | Virgin Islands (U.S.) | Latin America & Caribbean |
| 126 | Aruba | Latin America & Caribbean |  |  |  |
| 127 | Bahamas, The | Latin America & Caribbean |  |  |  |
| 128 | Barbados | Latin America & Caribbean |  |  |  |
| 129 | Belize | Latin America & Caribbean |  |  |  |
| 130 | Bolivia | Latin America & Caribbean |  |  |  |
| 131 | Brazil | Latin America & Caribbean |  |  |  |
| 132 | British Virgin Islands | Latin America & Caribbean |  |  |  |
| 133 | Cayman Islands | Latin America & Caribbean |  |  |  |
| 134 | Chile | Latin America & Caribbean |  |  |  |
| 135 | Colombia | Latin America & Caribbean |  |  |  |
| 136 | Costa Rica | Latin America & Caribbean |  |  |  |
| 137 | Cuba | Latin America & Caribbean |  |  |  |
| 138 | Curacao | Latin America & Caribbean |  |  |  |
| 139 | Dominica | Latin America & Caribbean |  |  |  |
| 140 | Dominican Republic | Latin America & Caribbean |  |  |  |
| 141 | Ecuador | Latin America & Caribbean |  |  |  |
| 142 | El Salvador | Latin America & Caribbean |  |  |  |
| 143 | Grenada | Latin America & Caribbean |  |  |  |
| 144 | Guatemala | Latin America & Caribbean |  |  |  |
| 145 | Guyana | Latin America & Caribbean |  |  |  |
| 146 | Haiti | Latin America & Caribbean |  |  |  |
| 147 | Honduras | Latin America & Caribbean |  |  |  |
| 148 | Jamaica | Latin America & Caribbean |  |  |  |
| 149 | Mexico | Latin America & Caribbean |  |  |  |
| 150 | Nicaragua | Latin America & Caribbean |  |  |  |
| 151 | Republic of Panama | Latin America & Caribbean |  |  |  |
| 152 | Panama | Latin America & Caribbean |  |  |  |
| 153 | Paraguay | Latin America & Caribbean |  |  |  |
| 154 | Peru | Latin America & Caribbean |  |  |  |
| 155 | Puerto Rico | Latin America & Caribbean |  |  |  |
| 156 | Sint Maarten (Dutch part) | Latin America & Caribbean |  |  |  |
| 157 | Saint Kitts and Nevis | Latin America & Caribbean |  |  |  |
| 158 | Saint Lucia | Latin America & Caribbean |  |  |  |
| 159 | St. Helena | Latin America & Caribbean |  |  |  |
| 160 | Saint Martin | Latin America & Caribbean |  |  |  |
| 161 | St. Vincent and the Grenadines | Latin America & Caribbean |  |  |  |
| 162 | Surinam | Latin America & Caribbean |  |  |  |
| 163 | Suriname | Latin America & Caribbean |  |  |  |
| 164 | Trinidad and Tobago | Latin America & Caribbean |  |  |  |
| 165 | Turks and Caicos Islands | Latin America & Caribbean |  |  |  |
| 166 | Uruguay | Latin America & Caribbean |  |  |  |

| Zone ne categories according to Qatar’s municiplaities system | | | |
| --- | --- | --- | --- |
| Zone Number | ZoneCateg | ZoneNumber | ZoneCateg |
| 0 | Urban | 49 | Urban |
| 1 | Urban | 50 | Urban |
| 2 | Urban | 51 | Urban |
| 3 | Urban | 52 | Urban |
| 4 | Urban | 53 | Urban |
| 5 | Urban | 54 | Urban |
| 6 | Urban | 55 | Urban |
| 7 | Urban | 56 | Urban |
| 10 | Urban | 57 | Urban |
| 11 | Urban | 58 | Urban |
| 12 | Urban | 60 | Urban |
| 13 | Urban | 61 | Urban |
| 14 | Urban | 62 | Urban |
| 15 | Urban | 63 | Urban |
| 16 | Urban | 64 | Urban |
| 17 | Urban | 65 | Urban |
| 18 | Urban | 66 | Urban |
| 19 | Urban | 67 | Urban |
| 20 | Urban | 68 | Urban |
| 21 | Urban | 69 | Urban |
| 22 | Urban | 70 | Rural |
| 23 | Urban | 71 | Rural |
| 24 | Urban | 72 | Rural |
| 25 | Urban | 73 | Rural |
| 26 | Urban | 74 | Rural |
| 27 | Urban | 75 | Rural |
| 28 | Urban | 76 | Rural |
| 29 | Urban | 77 | Rural |
| 30 | Urban | 78 | Rural |
| 31 | Urban | 79 | Rural |
| 32 | Urban | 80 | Rural |
| 33 | Urban | 81 | Rural |
| 34 | Urban | 82 | Rural |
| 35 | Urban | 83 | Rural |
| 36 | Urban | 84 | Rural |
| 37 | Urban | 85 | Rural |
| 38 | Urban | 86 | Rural |
| 39 | Urban | 90 | Rural |
| 40 | Urban | 91 | Rural |
| 41 | Urban | 92 | Rural |
| 42 | Urban | 93 | Rural |
| 43 | Urban | 94 | Rural |
| 44 | Urban | 95 | Rural |
| 45 | Urban | 96 | Rural |
| 46 | Urban | 97 | Rural |
| 47 | Urban | 98 | Rural |
| 48 | Urban | 0 | Urban |

| Emergency medical Dispatch chief complaint codes | | | | | | | | | | | |
| --- | --- | --- | --- | --- | --- | --- | --- | --- | --- | --- | --- |
| Dispatch Code | Chief Complaint | Dispatch Code | Chief Complaint | Dispatch Code | Chief Complaint | Dispatch Code | Chief Complaint | Dispatch Code | Chief Complaint | Dispatch Code | Chief Complaint |
| 01A01 | Abdominal Pain | 07D05 | Fire/Burn | 122800 | Seizure | 150801 | Electrocution/Lightening | 28C01G | Strock | 29D03U | RTA |
| 01A02 | Abdominal Pain | 07D05E | Fire/Burn | 122900 | Seizure | 150802 | Electrocution/Lightening | 28C01H | Strock | 29D03V | RTA |
| 01C00 | Abdominal Pain | 07D05F | Fire/Burn | 123200 | Seizure | 150803 | Electrocution/Lightening | 28C01J | Strock | 29D04 | RTA |
| 01C01 | Abdominal Pain | 08B01 | HazMat | 123201 | Seizure | 15C01E | Electrocution/Lightening | 28C01K | Strock | 29D04V | RTA |
| 01C02 | Abdominal Pain | 08B01C | HazMat | 123202 | Seizure | 15C01L | Electrocution/Lightening | 28C01L | Strock | 29D04Y | RTA |
| 01C03 | Abdominal Pain | 08B01G | HazMat | 123300 | Seizure | 15D02E | Electrocution/Lightening | 28C01M | Strock | 29D05 | RTA |
| 01C04 | Abdominal Pain | 08B01M | HazMat | 123400 | Seizure | 15D04E | Electrocution/Lightening | 28C01U | Strock | 29D05U | RTA |
| 01C05 | Abdominal Pain | 08B01U | HazMat | 123501 | Seizure | 15D07E | Electrocution/Lightening | 28C01X | Strock | 29D05V | RTA |
| 01C06 | Abdominal Pain | 08C01 | HazMat | 123502 | Seizure | 15D08E | Electrocution/Lightening | 28C01Y | Strock | 29D05X | RTA |
| 01D00 | Abdominal Pain | 08C01C | HazMat | 123601 | Seizure | 15D08L | Electrocution/Lightening | 28C01Z | Strock | 29D05Y | RTA |
| 01D01 | Abdominal Pain | 08C01G | HazMat | 123602 | Seizure | 15D09E | Electrocution/Lightening | 28C02C | Strock | 29D06 | RTA |
| 02A01 | Allergic reaction | 08C01M | HazMat | 123603 | Seizure | 15E01E | Electrocution/Lightening | 28C02F | Strock | 29D06U | RTA |
| 02A01I | Allergic reaction | 08C01U | HazMat | 123604 | Seizure | 160800 | Eye problem | 28C02G | Strock | 29D06V | RTA |
| 02A01M | Allergic reaction | 08D01G | HazMat | 123605 | Seizure | 160900 | Eye problem | 28C02H | Strock | 29D06Y | RTA |
| 02A02 | Allergic reaction | 08D02 | HazMat | 123700 | Seizure | 161100 | Eye problem | 28C02J | Strock | 29D07 | RTA |
| 02B00 | Allergic reaction | 08D02C | HazMat | 123800 | Seizure | 16A01 | Eye problem | 28C02K | Strock | 29D07U | RTA |
| 02B00M | Allergic reaction | 08D02G | HazMat | 123801 | Seizure | 16A02 | Eye problem | 28C02L | Strock | 29D07V | RTA |
| 02B01 | Allergic reaction | 08D02M | HazMat | 123802 | Seizure | 16A03 | Eye problem | 28C02U | Strock | 29D08 | RTA |
| 02B01I | Allergic reaction | 08D03C | HazMat | 124000 | Seizure | 16B00 | Eye problem | 28C02X | Strock | 29D08V | RTA |
| 02B01M | Allergic reaction | 08D03G | HazMat | 124200 | Seizure | 16B01 | Eye problem | 28C02Y | Strock | 29D09 | RTA |
| 02C01 | Allergic reaction | 08D03M | HazMat | 124201 | Seizure | 16D01 | Eye problem | 28C03C | Strock | 29D09U | RTA |
| 02C01I | Allergic reaction | 08D03U | HazMat | 124300 | Seizure | 170102 | Fall | 28C03D | Strock | 29D09V | RTA |
| 02C01M | Allergic reaction | 08D04 | HazMat | 124301 | Seizure | 170203 | Fall | 28C03F | Strock | 29D09Y | RTA |
| 02C02 | Allergic reaction | 08D04B | HazMat | 124302 | Seizure | 170501 | Fall | 28C03G | Strock | 29O01 | RTA |
| 02C02I | Allergic reaction | 08D04C | HazMat | 124600 | Seizure | 170502 | Fall | 28C03H | Strock | 29O01U | RTA |
| 02C02M | Allergic reaction | 08D04G | HazMat | 124700 | Seizure | 170503 | Fall | 28C03J | Strock | 29O01V | RTA |
| 02D01 | Allergic reaction | 08D04M | HazMat | 124800 | Seizure | 170506 | Fall | 28C03K | Strock | 29O01Y | RTA |
| 02D01I | Allergic reaction | 08D04T | HazMat | 124900 | Seizure | 170702 | Fall | 28C03L | Strock | 30A01 | Traumatic injury |
| 02D01M | Allergic reaction | 08D04U | HazMat | 125400 | Seizure | 170704 | Fall | 28C03U | Strock | 30A02 | Traumatic injury |
| 02D02 | Allergic reaction | 08D05 | HazMat | 125402 | Seizure | 170706 | Fall | 28C03X | Strock | 30A03 | Traumatic injury |
| 02D02I | Allergic reaction | 08D05B | HazMat | 125500 | Seizure | 170709 | Fall | 28C03Y | Strock | 30B00 | Traumatic injury |
| 02D02M | Allergic reaction | 08D05C | HazMat | 125600 | Seizure | 170902 | Fall | 28C04C | Strock | 30B01 | Traumatic injury |
| 02D03 | Allergic reaction | 08D05G | HazMat | 125800 | Seizure | 170908 | Fall | 28C04D | Strock | 30B02 | Traumatic injury |
| 02D04 | Allergic reaction | 08D05M | HazMat | 126200 | Seizure | 170909 | Fall | 28C04F | Strock | 30B03 | Traumatic injury |
| 02E01 | Allergic reaction | 08D05U | HazMat | 126300 | Seizure | 171101 | Fall | 28C04G | Strock | 30D01 | Traumatic injury |
| 03A01 | Animal attack/bite | 08D06C | HazMat | 126301 | Seizure | 171201 | Fall | 28C04H | Strock | 30D02 | Traumatic injury |
| 03A02 | Animal attack/bite | 08D06G | HazMat | 126303 | Seizure | 171202 | Fall | 28C04J | Strock | 30D03 | Traumatic injury |
| 03A03 | Animal attack/bite | 08D06T | HazMat | 126401 | Seizure | 171203 | Fall | 28C04K | Strock | 30D04 | Traumatic injury |
| 03A04 | Animal attack/bite | 09B01a | Cardiac arrest | 126500 | Seizure | 171205 | Fall | 28C04L | Strock | 30D05 | Traumatic injury |
| 03B00 | Animal attack/bite | 09B01b | Cardiac arrest | 126501 | Seizure | 171301 | Fall | 28C04U | Strock | 31A01 | Uncounscious |
| 03B01 | Animal attack/bite | 09B01c | Cardiac arrest | 126502 | Seizure | 171303 | Fall | 28C04X | Strock | 31A02 | Uncounscious |
| 03B02 | Animal attack/bite | 09B01e | Cardiac arrest | 126601 | Seizure | 171304 | Fall | 28C04Y | Strock | 31A03 | Uncounscious |
| 03B03 | Animal attack/bite | 09B01f | Cardiac arrest | 126602 | Seizure | 171305 | Fall | 28C05C | Strock | 31C00 | Uncounscious |
| 03D02 | Animal attack/bite | 09D01 | Cardiac arrest | 126803 | Seizure | 171406 | Fall | 28C05D | Strock | 31C01 | Uncounscious |
| 03D03 | Animal attack/bite | 09D02 | Cardiac arrest | 126900 | Seizure | 171601 | Fall | 28C05F | Strock | 31C02 | Uncounscious |
| 03D04 | Animal attack/bite | 09D02a | Cardiac arrest | 127000 | Seizure | 171602 | Fall | 28C05G | Strock | 31C03 | Uncounscious |
| 03D05 | Animal attack/bite | 09D02e | Cardiac arrest | 127200 | Seizure | 171603 | Fall | 28C05J | Strock | 31D01 | Uncounscious |
| 03D06 | Animal attack/bite | 09D02x | Cardiac arrest | 127300 | Seizure | 171604 | Fall | 28C05K | Strock | 31D02 | Uncounscious |
| 03D07 | Animal attack/bite | 09E01 | Cardiac arrest | 127400 | Seizure | 171605 | Fall | 28C05L | Strock | 31D03 | Uncounscious |
| 03D08 | Animal attack/bite | 09E02 | Cardiac arrest | 127600 | Seizure | 171606 | Fall | 28C05U | Strock | 31D04 | Uncounscious |
| 03D09 | Animal attack/bite | 09E03 | Cardiac arrest | 127700 | Seizure | 171608 | Fall | 28C05X | Strock | 31D05 | Uncounscious |
| 04A01A | Assault | 09E04 | Cardiac arrest | 127701 | Seizure | 171609 | Fall | 28C05Y | Strock | 31E00 | Uncounscious |
| 04A02A | Assault | 10A01 | Chest pain | 127800 | Seizure | 171701 | Fall | 28C06G | Strock | 31E01 | Uncounscious |
| 04A02S | Assault | 10C00 | Chest pain | 127901 | Seizure | 171703 | Fall | 28C06J | Strock | 32B01 | Unknown problem |
| 04A02T | Assault | 10C01 | Chest pain | 129100 | Seizure | 171705 | Fall | 28C06L | Strock | 32B02 | Unknown problem |
| 04A03A | Assault | 10C03 | Chest pain | 129500 | Seizure | 171706 | Fall | 28C06X | Strock | 32B03 | Unknown problem |
| 04A03S | Assault | 10D01 | Chest pain | 129600 | Seizure | 171709 | Fall | 28C06Y | Strock | 32B04 | Unknown problem |
| 04B00A | Assault | 10D02 | Chest pain | 129800 | Seizure | 171800 | Fall | 28C07C | Strock | 32D00 | Unknown problem |
| 04B00S | Assault | 10D03 | Chest pain | 129801 | Seizure | 171900 | Fall | 28C07D | Strock | 32D01 | Unknown problem |
| 04B01A | Assault | 10D04 | Chest pain | 129802 | Seizure | 172000 | Fall | 28C07F | Strock | 33A01P | IFT-Health Center |
| 04B01S | Assault | 10D05 | Chest pain | 12A01E | Seizure | 173001 | Fall | 28C07G | Strock | 33A01T | IFT-Health Center |
| 04B02A | Assault | 110101 | Chocking | 12A02 | Seizure | 173002 | Fall | 28C07H | Strock | 33A02T | IFT-Health Center |
| 04B03A | Assault | 110102 | Chocking | 12A03 | Seizure | 17A01 | Fall | 28C07J | Strock | 33A03T | IFT-Health Center |
| 04B03S | Assault | 110103 | Chocking | 12A03E | Seizure | 17A01A | Fall | 28C07L | Strock | 33C00T | IFT-Health Center |
| 04B03T | Assault | 110200 | Chocking | 12A04 | Seizure | 17A01G | Fall | 28C07X | Strock | 33C01T | IFT-Health Center |
| 04D00T | Assault | 1102601 | Chocking | 12A04E | Seizure | 17A01J | Fall | 28C07Y | Strock | 33C02T | IFT-Health Center |
| 04D01A | Assault | 110500 | Chocking | 12A05 | Seizure | 17A01P | Fall | 28C08C | Strock | 33C03T | IFT-Health Center |
| 04D02A | Assault | 110501 | Chocking | 12A05E | Seizure | 17A02 | Fall | 28C08G | Strock | 33C04T | IFT-Health Center |
| 04D03A | Assault | 110502 | Chocking | 12B00E | Seizure | 17A02A | Fall | 28C08J | Strock | 33C05T | IFT-Health Center |
| 04D03S | Assault | 110601 | Chocking | 12B01 | Seizure | 17A02E | Fall | 28C08L | Strock | 33C06T | IFT-Health Center |
| 04D04A | Assault | 110602 | Chocking | 12B01E | Seizure | 17A02G | Fall | 28C08X | Strock | 33C07T | IFT-Health Center |
| 04D05A | Assault | 110603 | Chocking | 12C00 | Seizure | 17A02P | Fall | 28C08Y | Strock | 33D00T | IFT-Health Center |
| 05A01 | Back pain | 110700 | Chocking | 12C01 | Seizure | 17A03 | Fall | 28C09C | Strock | 360002 | Pandemic |
| 05A02 | Back pain | 110800 | Chocking | 12C01E | Seizure | 17A03G | Fall | 28C09D | Strock | 36A01A | Pandemic |
| 05C00 | Back pain | 110802 | Chocking | 12C02 | Seizure | 17A04 | Fall | 28C09G | Strock | 36A01C | Pandemic |
| 05C01 | Back pain | 110901 | Chocking | 12C02E | Seizure | 17A04G | Fall | 28C09J | Strock | 36A01S | Pandemic |
| 05C02 | Back pain | 110902 | Chocking | 12C03 | Seizure | 17B00 | Fall | 28C09K | Strock | 36A02A | Pandemic |
| 05C03 | Back pain | 110903 | Chocking | 12C03E | Seizure | 17B00G | Fall | 28C09L | Strock | 36A02B | Pandemic |
| 05C04 | Back pain | 111100 | Chocking | 12C04 | Seizure | 17B00P | Fall | 28C09X | Strock | 36A02C | Pandemic |
| 05D01 | Back pain | 111200 | Chocking | 12C05 | Seizure | 17B01 | Fall | 28C10Z | Strock | 36A02S | Pandemic |
| 06C01 | Breathing problem | 111512 | Chocking | 12C05E | Seizure | 17B01G | Fall | 28C11C | Strock | 36A03A | Pandemic |
| 06C01A | Breathing problem | 111521 | Chocking | 12C07 | Seizure | 17B01J | Fall | 28C11G | Strock | 36A03B | Pandemic |
| 06C01E | Breathing problem | 111526 | Chocking | 12C07E | Seizure | 17B01P | Fall | 28C11L | Strock | 36A03C | Pandemic |
| 06C01O | Breathing problem | 111527 | Chocking | 12D01 | Seizure | 17B02 | Fall | 28C11U | Strock | 36A03S | Pandemic |
| 06C02 | Breathing problem | 11160 | Chocking | 12D01E | Seizure | 17B02G | Fall | 28C11X | Strock | 36A04 | Pandemic |
| 06C02A | Breathing problem | 111602 | Chocking | 12D02 | Seizure | 17B02P | Fall | 28C11Y | Strock | 36C00A | Pandemic |
| 06C02O | Breathing problem | 111702 | Chocking | 12D02E | Seizure | 17B03 | Fall | 28C12G | Strock | 36C00B | Pandemic |
| 06D01 | Breathing problem | 111901 | Chocking | 12D03 | Seizure | 17B03E | Fall | 28C12U | Strock | 36C00C | Pandemic |
| 06D01A | Breathing problem | 111902 | Chocking | 12D03E | Seizure | 17B03G | Fall | 29A00V | RTA | 36C00S | Pandemic |
| 06D01E | Breathing problem | 111903 | Chocking | 12D04 | Seizure | 17B03J | Fall | 29A00X | RTA | 36C01A | Pandemic |
| 06D01O | Breathing problem | 112001 | Chocking | 12D04E | Seizure | 17B03P | Fall | 29A01 | RTA | 36C01B | Pandemic |
| 06D02 | Breathing problem | 112003 | Chocking | 13A01 | Diabetic problem | 17B04 | Fall | 29A01V | RTA | 36C01C | Pandemic |
| 06D02A | Breathing problem | 1120031 | Chocking | 13C00 | Diabetic problem | 17B04G | Fall | 29A01Y | RTA | 36C01S | Pandemic |
| 06D02E | Breathing problem | 1120032 | Chocking | 13C01 | Diabetic problem | 17B04P | Fall | 29A02 | RTA | 36C02A | Pandemic |
| 06D02O | Breathing problem | 112101 | Chocking | 13C01C | Diabetic problem | 17D01 | Fall | 29A02U | RTA | 36C02B | Pandemic |
| 06D03 | Breathing problem | 112102 | Chocking | 13C02 | Diabetic problem | 17D01E | Fall | 29A02V | RTA | 36C02C | Pandemic |
| 06D03A | Breathing problem | 112202 | Chocking | 13C02C | Diabetic problem | 17D02 | Fall | 29A02X | RTA | 36C02S | Pandemic |
| 06D03E | Breathing problem | 112204 | Chocking | 13C03 | Diabetic problem | 17D03 | Fall | 29A02Y | RTA | 36C03A | Pandemic |
| 06D03O | Breathing problem | 112206 | Chocking | 13C03C | Diabetic problem | 17D04 | Fall | 29B00 | RTA | 36C03B | Pandemic |
| 06D04 | Breathing problem | 11A01C | Chocking | 13D01 | Diabetic problem | 17D04G | Fall | 29B00U | RTA | 36C03C | Pandemic |
| 06D04A | Breathing problem | 11A01F | Chocking | 140800 | Drowning/Near drowning | 17D04P | Fall | 29B01 | RTA | 36C03S | Pandemic |
| 06D04E | Breathing problem | 11A01M | Chocking | 14A01 | Drowning/Near drowning | 17D05 | Fall | 29B01U | RTA | 36C04A | Pandemic |
| 06D04O | Breathing problem | 11A01O | Chocking | 14A01S | Drowning/Near drowning | 17D05G | Fall | 29B01V | RTA | 36C04B | Pandemic |
| 06D05 | Breathing problem | 11A01U | Chocking | 14B01 | Drowning/Near drowning | 17D06 | Fall | 29B01X | RTA | 36C04C | Pandemic |
| 06E00 | Breathing problem | 11D01C | Chocking | 14B01D | Drowning/Near drowning | 180102 | Headache | 29B01Y | RTA | 36C04S | Pandemic |
| 06E00O | Breathing problem | 11D01F | Chocking | 14B01S | Drowning/Near drowning | 180400 | Headache | 29B02 | RTA | 36C05A | Pandemic |
| 06E01 | Breathing problem | 11D01M | Chocking | 14C01 | Drowning/Near drowning | 180502 | Headache | 29B02U | RTA | 36C05B | Pandemic |
| 06E01A | Breathing problem | 11D01O | Chocking | 14D01 | Drowning/Near drowning | 180503 | Headache | 29B02V | RTA | 36C05C | Pandemic |
| 06E01O | Breathing problem | 11D01U | Chocking | 14D01D | Drowning/Near drowning | 18A01 | Headache | 29B02Y | RTA | 36C05S | Pandemic |
| 07A01 | Fire/Burn | 11D02F | Chocking | 14D02 | Drowning/Near drowning | 18B00 | Headache | 29B03 | RTA | 36D00A | Pandemic |
| 07A01E | Fire/Burn | 11D02M | Chocking | 14D04 | Drowning/Near drowning | 18B01 | Headache | 29B03U | RTA | 36D00B | Pandemic |
| 07A01F | Fire/Burn | 11D02O | Chocking | 14D05 | Drowning/Near drowning | 18C00 | Headache | 29B03V | RTA | 36D00C | Pandemic |
| 07A02 | Fire/Burn | 11D02U | Chocking | 14D05D | Drowning/Near drowning | 18C00Y | Headache | 29B03X | RTA | 36D01A | Pandemic |
| 07A03 | Fire/Burn | 11E01 | Chocking | 14D05S | Drowning/Near drowning | 18C01 | Headache | 29B03Y | RTA | 36D01B | Pandemic |
| 07A03E | Fire/Burn | 11E01C | Chocking | 14E01 | Drowning/Near drowning | 18C01C | Headache | 29B04 | RTA | 36D01C | Pandemic |
| 07A04 | Fire/Burn | 11E01F | Chocking | 14E02 | Drowning/Near drowning | 18C01D | Headache | 29B04V | RTA | 36D01S | Pandemic |
| 07A05 | Fire/Burn | 11E01M | Chocking | 150101 | Electrocution/Lightening | 18C01F | Headache | 29B04X | RTA | 36D02A | Pandemic |
| 07B00 | Fire/Burn | 11E01O | Chocking | 150102 | Electrocution/Lightening | 18C01G | Headache | 29B04Y | RTA | 36D02B | Pandemic |
| 07B01 | Fire/Burn | 11E01U | Chocking | 150104 | Electrocution/Lightening | 18C01H | Headache | 29B05 | RTA | 36D02C | Pandemic |
| 07B01E | Fire/Burn | 120010 | Seizure | 150106 | Electrocution/Lightening | 18C01J | Headache | 29B05U | RTA | 36D02S | Pandemic |
| 07B02 | Fire/Burn | 1200101 | Seizure | 150108 | Electrocution/Lightening | 18C01K | Headache | 29B05V | RTA | 36D03A | Pandemic |
| 07B02E | Fire/Burn | 1200201 | Seizure | 150110 | Electrocution/Lightening | 18C01L | Headache | 29B05X | RTA | 36D03B | Pandemic |
| 07B02F | Fire/Burn | 1200202 | Seizure | 150201 | Electrocution/Lightening | 18C01U | Headache | 29B05Y | RTA | 36D03C | Pandemic |
| 07B02W | Fire/Burn | 120700 | Seizure | 150301 | Electrocution/Lightening | 18C01X | Headache | 29D00Y | RTA | 36D03S | Pandemic |
| 07C01F | Fire/Burn | 120800 | Seizure | 150302 | Electrocution/Lightening | 18C01Y | Headache | 29D01a | RTA | 36D04A | Pandemic |
| 07C02 | Fire/Burn | 121000 | Seizure | 150304 | Electrocution/Lightening | 18C01Z | Headache | 29D01b | RTA | 36D04B | Pandemic |
| 07C02E | Fire/Burn | 121100 | Seizure | 150305 | Electrocution/Lightening | 18C02 | Headache | 29D01d | RTA | 36D04C | Pandemic |
| 07C02F | Fire/Burn | 121200 | Seizure | 150311 | Electrocution/Lightening | 18C02C | Headache | 29D01e | RTA | 36D04S | Pandemic |
| 07C03 | Fire/Burn | 121300 | Seizure | 150313 | Electrocution/Lightening | 18C02D | Headache | 29D01f | RTA | 37B01 | IFT-Hospital |
| 07C03E | Fire/Burn | 121400 | Seizure | 150314 | Electrocution/Lightening | 18C02G | Headache | 29D01h | RTA | 37B01S | IFT-Hospital |
| 07C04 | Fire/Burn | 121500 | Seizure | 150503 | Electrocution/Lightening | 18C02H | Headache | 29D02k | RTA | 37B02 | IFT-Hospital |
| 07C04E | Fire/Burn | 121600 | Seizure | 150504 | Electrocution/Lightening | 18C02J | Headache | 29D02l | RTA | 37B02S | IFT-Hospital |
| 07D01 | Fire/Burn | 121602 | Seizure | 150505 | Electrocution/Lightening | 18C02K | Headache | 29D02m | RTA | 37C02 | IFT-Hospital |
| 07D01E | Fire/Burn | 121900 | Seizure | 150601 | Electrocution/Lightening | 18C02L | Headache | 29D02n | RTA | 37C04 | IFT-Hospital |
| 07D01F | Fire/Burn | 122100 | Seizure | 150602 | Electrocution/Lightening | 18C02M | Headache | 29D02o | RTA | 37C04B | IFT-Hospital |
| 07D02 | Fire/Burn | 122101 | Seizure | 150606 | Electrocution/Lightening | 18C02U | Headache | 29D02p | RTA | 37C05 | IFT-Hospital |
| 07D02F | Fire/Burn | 122102 | Seizure | 150608 | Electrocution/Lightening | 18C02X | Headache | 29D02q | RTA | 37C05A | IFT-Hospital |
| 07D04 | Fire/Burn | 122300 | Seizure | 150611 | Electrocution/Lightening | 18C02Y | Headache | 29D02r | RTA | AS10X | Walking patient |
| 07D04E | Fire/Burn | 122400 | Seizure | 150612 | Electrocution/Lightening | 18C03C | Headache | 29D02s | RTA | AS10Y | Walking patient |
| 07D04F | Fire/Burn | 122500 | Seizure | 150613 | Electrocution/Lightening | 18C03D | Headache | 29D02t | RTA | AS12X | Walking patient |
|  |  | 122600 | Seizure | 150700 | Electrocution/Lightening | 18C03F | Headache | 29D03 | RTA | AS12Y | Walking patient |
| Dispatch Code | **Chief Complaint** | **Dispatch Code** | **Chief Complaint** | **Dispatch Code** | **Chief Complaint** | **Dispatch Code** | **Chief Complaint** | **Dispatch Code** | **Chief Complaint** | **Dispatch Code** | **Chief Complaint** |
| 18C03G | Headache | 190800 | Heart problems | 23C02I | Poisoning | 26A00 | Sick person |  |  | AS14X | Walking patient |
| 18C03H | Headache | 191700 | Heart problems | 23C02V | Poisoning | 26A01 | Sick person |  |  | AS15X | Walking patient |
| 18C03J | Headache | 191800 | Heart problems | 23C03A | Poisoning | 26A02 | Sick person |  |  | AS16X | Walking patient |
| 18C03K | Headache | 192100 | Heart problems | 23C03I | Poisoning | 26A03 | Sick person |  |  | AS17X | Walking patient |
| 18C03L | Headache | 193400 | Heart problems | 23C04A | Poisoning | 26A04 | Sick person |  |  | AS17Y | Walking patient |
| 18C03U | Headache | 19A01 | Heart problems | 23C04I | Poisoning | 26A05 | Sick person |  |  | AS18X | Walking patient |
| 18C03X | Headache | 19A02 | Heart problems | 23C06A | Poisoning | 26A06 | Sick person |  |  | AS18Y | Walking patient |
| 18C03Y | Headache | 19C00 | Heart problems | 23C06I | Poisoning | 26A07 | Sick person |  |  | AS19X | Walking patient |
| 18C03Z | Headache | 19C01 | Heart problems | 23C07A | Poisoning | 26A08 | Sick person |  |  | AS19Y | Walking patient |
| 18C04C | Headache | 19C02 | Heart problems | 23C07I | Poisoning | 26A09 | Sick person |  |  | AS1X | Walking patient |
| 18C04D | Headache | 19C03 | Heart problems | 23C07V | Poisoning | 26A10 | Sick person |  |  | AS1Y | Walking patient |
| 18C04F | Headache | 19C04 | Heart problems | 23C07W | Poisoning | 26A11 | Sick person |  |  | AS20X | Walking patient |
| 18C04G | Headache | 19C06 | Heart problems | 23C08 | Poisoning | 26A12 | Sick person |  |  | AS21X | Walking patient |
| 18C04H | Headache | 19C07 | Heart problems | 23D01A | Poisoning | 26B00 | Sick person |  |  | AS22X | Walking patient |
| 18C04J | Headache | 19D01 | Heart problems | 23D01I | Poisoning | 26B01 | Sick person |  |  | AS23X | Walking patient |
| 18C04K | Headache | 19D02 | Heart problems | 23D02A | Poisoning | 26C00 | Sick person |  |  | AS24X | Walking patient |
| 18C04L | Headache | 19D03 | Heart problems | 23D02I | Poisoning | 26C01 | Sick person |  |  | AS25X | Walking patient |
| 18C04U | Headache | 19D04 | Heart problems | 23D03A | Poisoning | 26C02 | Sick person |  |  | AS25Y | Walking patient |
| 18C04X | Headache | 200010 | Heat related | 23D03I | Poisoning | 26C03 | Sick person |  |  | AS26X | Walking patient |
| 18C04Y | Headache | 200011 | Heat related | 23O01A | Poisoning | 26C04 | Sick person |  |  | AS26Y | Walking patient |
| 18C04Z | Headache | 200999 | Heat related | 24A01 | Pregnancy | 26D00 | Sick person |  |  | AS29X | Walking patient |
| 18C05D | Headache | 203800 | Heat related | 24B01 | Pregnancy | 26D01 | Sick person |  |  | AS29Y | Walking patient |
| 18C05G | Headache | 203901 | Heat related | 24B01M | Pregnancy | 26O02 | Sick person |  |  | AS2X | Walking patient |
| 18C05H | Headache | 204000 | Heat related | 24B02 | Pregnancy | 26O03 | Sick person |  |  | AS2Y | Walking patient |
| 18C05J | Headache | 20A01C | Heat related | 24C01 | Pregnancy | 26O04 | Sick person |  |  | AS30X | Walking patient |
| 18C05K | Headache | 20A01H | Heat related | 24C02 | Pregnancy | 26O05 | Sick person |  |  | AS30Y | Walking patient |
| 18C05L | Headache | 20B00H | Heat related | 24C03 | Pregnancy | 26O06 | Sick person |  |  | AS31X | Walking patient |
| 18C05U | Headache | 20B01H | Heat related | 24C03M | Pregnancy | 26O07 | Sick person |  |  | AS31Y | Walking patient |
| 18C05X | Headache | 20B02C | Heat related | 24D03 | Pregnancy | 26O08 | Sick person |  |  | AS32X | Walking patient |
| 18C05Y | Headache | 20B02H | Heat related | 24D03M | Pregnancy | 26O09 | Sick person |  |  | AS32Y | Walking patient |
| 18C06G | Headache | 20C01H | Heat related | 24D04 | Pregnancy | 26O10 | Sick person |  |  | AS3X | Walking patient |
| 18C06J | Headache | 20D01C | Heat related | 24D05 | Pregnancy | 26O11 | Sick person |  |  | AS4X | Walking patient |
| 18C06L | Headache | 20D01H | Heat related | 24D05M | Pregnancy | 26O12 | Sick person |  |  | AS4Y | Walking patient |
| 18C06X | Headache | 20D02H | Heat related | 24O01 | Pregnancy | 26O13 | Sick person |  |  | AS5X | Walking patient |
| 18C06Y | Headache | 210601 | Bleeding | 250103 | Abnormal behaviour | 26O14 | Sick person |  |  | AS6X | Walking patient |
| 18C07D | Headache | 2121243 | Bleeding | 250111 | Abnormal behaviour | 26O15 | Sick person |  |  | AS6Y | Walking patient |
| 18C07F | Headache | 21A01M | Bleeding | 250203 | Abnormal behaviour | 26O16 | Sick person |  |  | AS7X | Walking patient |
| 18C07G | Headache | 21A01T | Bleeding | 250401 | Abnormal behaviour | 26O17 | Sick person |  |  | AS7Y | Walking patient |
| 18C07K | Headache | 21A02M | Bleeding | 25A01 | Abnormal behaviour | 26O18 | Sick person |  |  | AS9X | Walking patient |
| 18C07L | Headache | 21A02T | Bleeding | 25A01B | Abnormal behaviour | 26O19 | Sick person |  |  | AS9Y | Walking patient |
| 18C07X | Headache | 21B00M | Bleeding | 25A01V | Abnormal behaviour | 26O20 | Sick person |  |  | ASX | Walking patient |
| 18C07Y | Headache | 21B00T | Bleeding | 25A01W | Abnormal behaviour | 26O21 | Sick person |  |  | ASY | Walking patient |
| 19002 | Heart problems | 21B01M | Bleeding | 25A02 | Abnormal behaviour | 26O22 | Sick person |  |  |  |  |
| 190100 | Heart problems | 21B01T | Bleeding | 25A02V | Abnormal behaviour | 26O23 | Sick person |  |  |  |  |
| 190200 | Heart problems | 21B02M | Bleeding | 25B02 | Abnormal behaviour | 26O24 | Sick person |  |  |  |  |
|  |  | 21B02T | Bleeding | 25B02B | Abnormal behaviour | 26O26 | Sick person |  |  |  |  |
|  |  | 21B03M | Bleeding | 25B02V | Abnormal behaviour | 26O27 | Sick person |  |  |  |  |
|  |  | 21B03T | Bleeding | 25B02W | Abnormal behaviour | 26O28 | Sick person |  |  |  |  |
|  |  | 21B04M | Bleeding | 25B03 | Abnormal behaviour | 27B02G | Stabbing/Gunshot |  |  |  |  |
|  |  | 21B04T | Bleeding | 25B03B | Abnormal behaviour | 27B02I | Stabbing/Gunshot |  |  |  |  |
|  |  | 21C01M | Bleeding | 25B03T | Abnormal behaviour | 27B02P | Stabbing/Gunshot |  |  |  |  |
|  |  | 21C01T | Bleeding | 25B03V | Abnormal behaviour | 27B02S | Stabbing/Gunshot |  |  |  |  |
|  |  | 21C02M | Bleeding | 25B03W | Abnormal behaviour | 27B02Y | Stabbing/Gunshot |  |  |  |  |
|  |  | 21C03M | Bleeding | 25B04 | Abnormal behaviour | 27B04S | Stabbing/Gunshot |  |  |  |  |
|  |  | 21C03T | Bleeding | 25B04V | Abnormal behaviour | 27D01S | Stabbing/Gunshot |  |  |  |  |
|  |  | 21D02M | Bleeding | 25B05 | Abnormal behaviour | 27D01Y | Stabbing/Gunshot |  |  |  |  |
|  |  | 21D02T | Bleeding | 25B06 | Abnormal behaviour | 27D02S | Stabbing/Gunshot |  |  |  |  |
|  |  | 21D03M | Bleeding | 25B06B | Abnormal behaviour | 27D03S | Stabbing/Gunshot |  |  |  |  |
|  |  | 21D03T | Bleeding | 25B06V | Abnormal behaviour | 27D03Y | Stabbing/Gunshot |  |  |  |  |
|  |  | 21D04M | Bleeding | 25B06W | Abnormal behaviour | 27D04S | Stabbing/Gunshot |  |  |  |  |
|  |  | 21D04T | Bleeding | 25D01 | Abnormal behaviour | 27D04Y | Stabbing/Gunshot |  |  |  |  |
|  |  | 21D05M | Bleeding | 25D01B | Abnormal behaviour | 27D05S | Stabbing/Gunshot |  |  |  |  |
|  |  | 21D05T | Bleeding | 25D01V | Abnormal behaviour | 27D05Y | Stabbing/Gunshot |  |  |  |  |
|  |  | 22A01 | Entrapement | 25D02 | Abnormal behaviour | 27D06S | Stabbing/Gunshot |  |  |  |  |
|  |  | 22A01M | Entrapement | 25D03 | Abnormal behaviour | 28A01G | Strock |  |  |  |  |
|  |  | 22B00 | Entrapement | 25D03B | Abnormal behaviour | 28A01J | Strock |  |  |  |  |
|  |  | 22B01 | Entrapement | 25D03V | Abnormal behaviour | 28A01L | Strock |  |  |  |  |
|  |  | 22B01B | Entrapement | 25D03W | Abnormal behaviour | 28A01U | Strock |  |  |  |  |
|  |  | 22B02 | Entrapement | 25D04 | Abnormal behaviour | 28A01X | Strock |  |  |  |  |
|  |  | 22B02A | Entrapement |  |  | 28C01C | Strock |  |  |  |  |
|  |  | 22B03 | Entrapement |  |  | 28C01D | Strock |  |  |  |  |
|  |  | 22B03A | Entrapement |  |  | 28C01F | Strock |  |  |  |  |
|  |  | 22D01 | Entrapement |  |  |  |  |  |  |  |  |
|  |  | 22D01A | Entrapement |  |  |  |  |  |  |  |  |
|  |  | 22D01M | Entrapement |  |  |  |  |  |  |  |  |
|  |  | 22D01X | Entrapement |  |  |  |  |  |  |  |  |
|  |  | 22D01Y | Entrapement |  |  |  |  |  |  |  |  |
|  |  | 22D03A | Entrapement |  |  |  |  |  |  |  |  |
|  |  | 22D04 | Entrapement |  |  |  |  |  |  |  |  |
|  |  | 22D04A | Entrapement |  |  |  |  |  |  |  |  |
|  |  | 22D04M | Entrapement |  |  |  |  |  |  |  |  |
|  |  | 22D04X | Entrapement |  |  |  |  |  |  |  |  |
|  |  | 22D05 | Entrapement |  |  |  |  |  |  |  |  |
|  |  | 22D05A | Entrapement |  |  |  |  |  |  |  |  |
|  |  | 22D05M | Entrapement |  |  |  |  |  |  |  |  |
|  |  | 230102 | Poisoning |  |  |  |  |  |  |  |  |
|  |  | 230701 | Poisoning |  |  |  |  |  |  |  |  |
|  |  | 23B01I | Poisoning |  |  |  |  |  |  |  |  |
|  |  | 23B01V | Poisoning |  |  |  |  |  |  |  |  |
|  |  | 23C01A | Poisoning |  |  |  |  |  |  |  |  |
|  |  | 23C01I | Poisoning |  |  |  |  |  |  |  |  |
|  |  | 23C01V | Poisoning |  |  |  |  |  |  |  |  |
|  |  | 23C02 | Poisoning |  |  |  |  |  |  |  |  |
|  |  | 23C02A | Poisoning |  |  |  |  |  |  |  |  |

| Responding unit categorisation | | | | | | | | | |
| --- | --- | --- | --- | --- | --- | --- | --- | --- | --- |
| Responding Unit | Type | Responding Unit | Type | Responding Unit | Type | Responding Unit | Type | Responding Unit | Type |
| 1.015 | Alpha | A5.025 | Alpha | b4.1 | Bravo | E-AL WAJBA | Event | HAZMAT 1 | SEM |
| 2.011 | Alpha | A5.026 | Alpha | BRV2.1 | Bravo | E-Asayel | Event | HAZMAT 2 | SEM |
| 2.02 | Alpha | A5.03 | Alpha | BRV4.1 | Bravo | E-ASPIRE | Event | HAZMAT 4 | SEM |
| 4.015 | Alpha | A5.04 | Alpha | BRV8.1 | Bravo | ECAMEL | Event | HAZMAT 4.01 | SEM |
| 4.10 | Alpha | A5.05 | Alpha | BRV8.2 | Bravo | E-CAMEL | Event | HAZMAT 4.1 | SEM |
| 5.001 | Alpha | A5.06 | Alpha | BRV8.3 | Bravo | e-camel3 | Event | HAZMAT 5 | SEM |
| 5.11 | Alpha | A5.07 | Alpha | BRV8.4 | Bravo | E-CAMPING 1 | Event | HAZMAT4.1 | SEM |
| A 7.011 | Alpha | A5.08 | Alpha | BRV8.5 | Bravo | e-cherch | Event | MIR 2.01 | SEM |
| a? | Alpha | A5.09 | Alpha | BRV8.6 | Bravo | e-church | Event | MIR 2.1 | SEM |
| A1.01 | Alpha | A5.1 | Alpha | BRV8.7 | Bravo | e-cup4 | Event | MIR 6.1 | SEM |
| A1.010 | Alpha | A5.10 | Alpha | BRV8.8 | Bravo | E-CYCLING 2 | Event | MIR4.01 | SEM |
| A1.011 | Alpha | A5.11 | Alpha | GOLF81 | Bravo | E-D7.1 | Event | MIR4.1 | SEM |
| A1.014 | Alpha | A5.12 | Alpha | GOLF82 | Bravo | E-D71 | Event | MIR6.1 | SEM |
| A1.02 | Alpha | A5.13 | Alpha | CDO | Charlie | E-DELTA 10 | Event | MIR7.01 | SEM |
| A1.03 | Alpha | A5.14 | Alpha | CH01 | Charlie | E-DRAG 1 | Event | MIR7.1 | SEM |
| A1.04 | Alpha | A5.15 | Alpha | CH02 | Charlie | E-DRAG 2 | Event | T 6.1 | Tango |
| A1.05 | Alpha | A5.16 | Alpha | CH03 | Charlie | E-DRAG 3 | Event | T1.10 | Tango |
| A1.06 | Alpha | A5.17 | Alpha | CH04 | Charlie | e-drag race2 | Event | T1.11 | Tango |
| A1.07 | Alpha | A5.18 | Alpha | CH05 | Charlie | edrag1 | Event | T1.12 | Tango |
| A1.08 | Alpha | A5.19 | Alpha | CH06 | Charlie | E-ENDURANCE | Event | T1.13 | Tango |
| A1.09 | Alpha | A5.2 | Alpha | CH07 | Charlie | E-EXHIBITION | Event | T1.14 | Tango |
| A1.1 | Alpha | A5.20 | Alpha | CH08 | Charlie | E-exibition | Event | T1.15 | Tango |
| A1.10 | Alpha | A5.21 | Alpha | CH09 | Charlie | E-fes 1 | Event | T1.16 | Tango |
| A1.11 | Alpha | A5.22 | Alpha | CH11 | Charlie | EFESTIVAL | Event | T1.17 | Tango |
| A1.12 | Alpha | A5.23 | Alpha | CH2.01 | Charlie | e-festival | Event | T1.18 | Tango |
| A1.13 | Alpha | A5.24 | Alpha | CH3.01 | Charlie | E-FESTIVAL 1 | Event | T1.19 | Tango |
| A1.14 | Alpha | A5.25 | Alpha | CH4.01 | Charlie | E-FESTIVAL 2 | Event | T1.2 | Tango |
| A1.15 | Alpha | A5.26 | Alpha | CH5.01 | Charlie | E-FIFA 1 | Event | T1.20 | Tango |
| A1.16 | Alpha | A5.27 | Alpha | CH7.01 | Charlie | E-FIFA 1.1 | Event | T1.21 | Tango |
| A1.17 | Alpha | A5.3 | Alpha | CHLF101 | Charlie | E-FOOTBALL 1 | Event | T1.22 | Tango |
| A1.18 | Alpha | A5.4 | Alpha | CHLF102 | Charlie | e-halal | Event | T1.23 | Tango |
| A1.19 | Alpha | A5.5 | Alpha | CHLF103 | Charlie | e-hayat | Event | T1.24 | Tango |
| A1.2 | Alpha | A5.6 | Alpha | COHORT 4 | Charlie | E-HORSE 1 | Event | T1.27 | Tango |
| A1.20 | Alpha | A5.7 | Alpha | COHORT 6 | Charlie | ekatara | Event | T1.28 | Tango |
| A1.21 | Alpha | A5.8 | Alpha | CVC | Charlie | e-katara | Event | T1.29 | Tango |
| A1.22 | Alpha | A5.9 | Alpha | LF101 | Charlie | e-katara1 | Event | T1.3 | Tango |
| A1.3 | Alpha | A6.01 | Alpha | LF102 | Charlie | Elousil | Event | T1.5 | Tango |
| A1.4 | Alpha | A6.010 | Alpha | LF103 | Charlie | e-lusail | Event | T1.7 | Tango |
| A1.5 | Alpha | A6.015 | Alpha | OSC. E | Charlie | E-mahamyl | Event | T1.8 | Tango |
| A1.6 | Alpha | A6.02 | Alpha | Qatar 1 | COVID | E-mahanil | Event | T1.9 | Tango |
| A1.7 | Alpha | A6.03 | Alpha | QATAR 1.4 | COVID | e-mahaseel | Event | T2.1 | Tango |
| A1.8 | Alpha | A6.04 | Alpha | QATAR 2 | COVID | e-majaheem | Event | T2.2 | Tango |
| A1.9 | Alpha | A6.05 | Alpha | Qatar 3 | COVID | E-MARATHON 1 | Event | T2.3 | Tango |
| A2.0.2 | Alpha | A6.06 | Alpha | QATAR 4.3 | COVID | E-MARATHON 3 | Event | T4.1 | Tango |
| A2.01 | Alpha | A6.07 | Alpha | QATAR 7 | COVID | emariot | Event | T4.2 | Tango |
| A2.010 | Alpha | A6.08 | Alpha | qatar1 | COVID | entretient | Event | T4.3 | Tango |
| A2.011 | Alpha | A6.09 | Alpha | QATAR1.02 | COVID | E-ORYX 2 | Event | T4.4 | Tango |
| A2.012 | Alpha | A6.1 | Alpha | QATAR1.1 | COVID | E-ORYX 3 | Event | T4.5 | Tango |
| A2.013 | Alpha | A6.10 | Alpha | QATAR1.2 | COVID | E-ORYX 4 | Event | T4.6 | Tango |
| A2.014 | Alpha | A6.11 | Alpha | QATAR1.8 | COVID | E-ORYX 5 | Event | T4.7 | Tango |
| A2.016 | Alpha | A6.12 | Alpha | QATAR10 | COVID | E-ORYX 6 | Event | T5.1 | Tango |
| A2.02 | Alpha | A6.13 | Alpha | qatar3 | COVID | E-Qatar 1 | Event | T5.2 | Tango |
| A2.03 | Alpha | A6.14 | Alpha | qatar4 | COVID | E-Qatar 3 | Event | T5.3 | Tango |
| A2.04 | Alpha | A6.15 | Alpha | QATAR5.4 | COVID | E-QATAR 4 | Event | T5.4 | Tango |
| A2.05 | Alpha | A6.18 | Alpha | QATAR5.5 | COVID | E-QATAR 4.3 | Event | T5.5 | Tango |
| A2.06 | Alpha | A6.2 | Alpha | QATAR5.6 | COVID | eqatara | Event | T7.1 | Tango |
| A2.07 | Alpha | A6.3 | Alpha | QATAR5.7 | COVID | E-QMMF | Event | T7.2 | Tango |
| A2.08 | Alpha | A6.4 | Alpha | QATAR6 | COVID | e-rail4 | Event |  |  |
| A2.09 | Alpha | A6.5 | Alpha | QATAR6.1 | COVID | E-SCHOOL | Event |  |  |
| A2.1 | Alpha | A6.6 | Alpha | QATAR7 | COVID | e-shanglrila | Event |  |  |
| A2.10 | Alpha | A6.7 | Alpha | QATAR8 | COVID | E-shangrila | Event |  |  |
| A2.11 | Alpha | A6.8 | Alpha | QATAR9 | COVID | E-SHAQAB 1 | Event |  |  |
| A2.12 | Alpha | A6.9 | Alpha | qx1 | COVID | E-SHERATON | Event |  |  |
| A2.13 | Alpha | A7.01 | Alpha | D 1 | Delta | ESOUK WAKEF | Event |  |  |
| A2.14 | Alpha | A7.010 | Alpha | D 10 | Delta | E-SOUK WKA | Event |  |  |
| A2.15 | Alpha | A7.02 | Alpha | D 2 | Delta | ESOUKWAKEF | Event |  |  |
| A2.16 | Alpha | A7.03 | Alpha | D 2.1 | Delta | E-souq | Event |  |  |
| A2.17 | Alpha | A7.04 | Alpha | D 3 | Delta | E-SPARTAN 2 | Event |  |  |
| A2.18 | Alpha | A7.05 | Alpha | D 4 | Delta | E-spring | Event |  |  |
| A2.19 | Alpha | A7.06 | Alpha | D 4.1 | Delta | E-UNIVERSITY | Event |  |  |
| A2.2 | Alpha | A7.07 | Alpha | D 5 | Delta | event | Event |  |  |
| A2.20 | Alpha | A7.08 | Alpha | D 5.1 | Delta | e-village | Event |  |  |
| A2.21 | Alpha | A7.09 | Alpha | D 6 | Delta | E-WAJBA | Event |  |  |
| A2.3 | Alpha | A7.1 | Alpha | D 6.1 | Delta | Exhibition | Event |  |  |
| A2.4 | Alpha | A7.10 | Alpha | D 7 | Delta | exibition unit | Event |  |  |
| A2.5 | Alpha | A7.11 | Alpha | D C 4 | Delta | FEVER-CLINIC 1 | Event |  |  |
| A2.6 | Alpha | A7.12 | Alpha | D10 | Delta | FEVER-CLINIC 2 | Event |  |  |
| A2.7 | Alpha | A7.13 | Alpha | D2 | Delta | FEVER-CLINIC 3 | Event |  |  |
| A2.8 | Alpha | A7.14 | Alpha | D4 | Delta | HMC1 | Event |  |  |
| A2.9 | Alpha | A7.15 | Alpha | D5.1 | Delta | katara | Event |  |  |
| A4.01 | Alpha | A7.16 | Alpha | D7 | Delta | ORYX 2 | Event |  |  |
| A4.010 | Alpha | A7.17 | Alpha | D9 | Delta | Rehersal 1 | Event |  |  |
| A4.011 | Alpha | A7.18 | Alpha | ajyal event | Event | samla2 | Event |  |  |
| A4.012 | Alpha | a7.19 | Alpha | almajahin | Event | VACCINE 1 | Event |  |  |
| A4.013 | Alpha | A7.2 | Alpha | aq1 | Event | VACCINE 1.01 | Event |  |  |
| A4.014 | Alpha | a7.20 | Alpha | assayel1 | Event | VACCINE 2 | Event |  |  |
| A4.015 | Alpha | a7.21 | Alpha | at7.23 | Event | VACCINE 3 | Event |  |  |
| A4.016 | Alpha | a7.22 | Alpha | at7.24 | Event | vaccine1.01 | Event |  |  |
| A4.02 | Alpha | A7.24 | Alpha | drag1 | Event | F1.01 | Foxtrot |  |  |
| A4.03 | Alpha | A7.25 | Alpha | Duhail 3 | Event | F1.012 | Foxtrot |  |  |
| A4.04 | Alpha | A7.26 | Alpha | E - A7.12 | Event | F1.013 | Foxtrot |  |  |
| A4.05 | Alpha | A7.27 | Alpha | E - A7.13 | Event | F1.02 | Foxtrot |  |  |
| A4.06 | Alpha | A7.3 | Alpha | E - A7.14 | Event | F1.03 | Foxtrot |  |  |
| A4.07 | Alpha | A7.4 | Alpha | E - A7.15 | Event | F1.05 | Foxtrot |  |  |
| A4.08 | Alpha | A7.5 | Alpha | E - A7.16 | Event | F1.06 | Foxtrot |  |  |
| A4.09 | Alpha | A7.6 | Alpha | E - A7.17 | Event | F1.1 | Foxtrot |  |  |
| A4.1 | Alpha | A7.7 | Alpha | E - A7.18 | Event | F1.10 | Foxtrot |  |  |
| A4.10 | Alpha | A7.8 | Alpha | E - A7.19 | Event | F1.11 | Foxtrot |  |  |
| A4.11 | Alpha | A7.9 | Alpha | E - A7.20 | Event | F1.12 | Foxtrot |  |  |
| A4.12 | Alpha | A8.1 | Alpha | E - A7.21 | Event | F1.13 | Foxtrot |  |  |
| A4.13 | Alpha | A8.2 | Alpha | E - A7.22 | Event | F1.14 | Foxtrot |  |  |
| A4.14 | Alpha | A8.3 | Alpha | E- A7.15 | Event | F1.15 | Foxtrot |  |  |
| A4.15 | Alpha | A8.4 | Alpha | E CAMEL | Event | F1.16 | Foxtrot |  |  |
| A4.16 | Alpha | DRIVING 1 | Alpha | E Camel2 | Event | F1.17 | Foxtrot |  |  |
| A4.17 | Alpha | DRIVING 2 | Alpha | e exhibition | Event | F1.2 | Foxtrot |  |  |
| A4.18 | Alpha | DRIVING 3 | Alpha | E EZDAN 4 | Event | F1.3 | Foxtrot |  |  |
| A4.19 | Alpha | DRIVING 4 | Alpha | E HORSE2 | Event | F1.4 | Foxtrot |  |  |
| A4.2 | Alpha | DRIVING 5 | Alpha | e katara | Event | F1.5 | Foxtrot |  |  |
| A4.20 | Alpha | DRIVING 6 | Alpha | E KATARA3 | Event | F1.6 | Foxtrot |  |  |
| A4.21 | Alpha | DRIVING 7 | Alpha | E LOUSIL | Event | F1.7 | Foxtrot |  |  |
| A4.22 | Alpha | driving2 | Alpha | e lusail | Event | F1.8 | Foxtrot |  |  |
| A4.23 | Alpha | driving4 | Alpha | e mahaseel | Event | F1.9 | Foxtrot |  |  |
| A4.24 | Alpha | LVQ01 | Alpha | E RAS GAZ | Event | F4.1 | Foxtrot |  |  |
| A4.25 | Alpha | LVQ1 | Alpha | E SCHOOL | Event | F6.01 | Foxtrot |  |  |
| A4.26 | Alpha | LVQ2 | Alpha | e souq wakra | Event | F6.1 | Foxtrot |  |  |
| A4.27 | Alpha | LVQ3 | Alpha | E Vaccin 1.01 | Event | F7.01 | Foxtrot |  |  |
| A4.28 | Alpha | POOL UNIT 1 | Alpha | E-A7.12 | Event | F7.1 | Foxtrot |  |  |
| A4.3 | Alpha |  |  | E-A7.12 4x4 Unit | Event | RETRIEVAL 1 | Foxtrot |  |  |
| A4.4 | Alpha |  |  | e-a7.13 | Event | RETRIEVAL 2 | Foxtrot |  |  |
| A4.5 | Alpha |  |  | E-A7.13 4X4 Unit | Event | gb 9 | Green bus |  |  |
| A4.6 | Alpha |  |  | E-A7.14 4X4 Unit | Event | GB0 | Green bus |  |  |
| A4.7 | Alpha |  |  | e-a7.15 | Event | GB5.1 | Green bus |  |  |
| A4.8 | Alpha |  |  | e-a7.16 | Event | gb6 | Green bus |  |  |
| A4.9 | Alpha |  |  | E-A7.17 | Event | GB7.1 | Green bus |  |  |
| A5.00 | Alpha |  |  | E-A7.18 | Event | GB8.1 | Green bus |  |  |
| A5.001 | Alpha |  |  | E-A7.22 | Event | GREEN BUS 2 | Green bus |  |  |
| A5.01 | Alpha |  |  | E-A712 | Event | GREEN BUS ZERO | Green bus |  |  |
| A5.010 | Alpha |  |  | E-A713 | Event | GREENBUS 0 | Green bus |  |  |
| A5.011 | Alpha |  |  | E-A714 | Event | GREENBUS 6 | Green bus |  |  |
| A5.012 | Alpha |  |  | E-A715 | Event | mike2 | Other |  |  |
| A5.013 | Alpha |  |  | E-A716 | Event | mile1 | Other |  |  |
| A5.014 | Alpha |  |  | E-A717 | Event | PRODUCTION MIR | Other |  |  |
| A5.015 | Alpha |  |  | E-A718 | Event | T-HAZMAT5 | Other |  |  |
| A5.016 | Alpha |  |  | E-A719 | Event | T-LOGISTIC5 | Other |  |  |
| A5.017 | Alpha |  |  | E-A720 | Event | X1A | Other |  |  |
| A5.019 | Alpha |  |  | E-A721 | Event | XI4 | Other |  |  |
| A5.02 | Alpha |  |  | E-A722 | Event | HAZM 1 | SEM |  |  |
| A5.021 | Alpha |  |  | E-AFC 11 | Event | HAZM 2 | SEM |  |  |
| A5.023 | Alpha |  |  | E-AFC 6 | Event | HAZM 3 | SEM |  |  |
| A5.024 | Alpha |  |  | E-AL NASHAAB 2 | Event | HAZM2 | SEM |  |  |

| Provisional diagnosis classification | | | |
| --- | --- | --- | --- |
| Provisional Diagnosis | Classification | Provisional Diagnosis | Classification |
| Allergies | Allergic reaction | CVA | Neurological |
| Other : Allergic Reaction | Allergic reaction | Neurological : Acute Stroke < 8 Hours | Neurological |
| Other : Anaphylaxis | Anaphylaxis | Neurological : Headache | Neurological |
| Animal Bite | Animal Bite | Neurological : Psychiatric Event | Neurological |
| Airway burns- Thermaldsad | Burns | Neurological : Reduced Level of Conciousness Unkno | Neurological |
| Burns | Burns | Neurological : Reduced Level of Conciousness Unknown Cause | Neurological |
| Burns : Chemical | Burns | Neurological : Seizure | Neurological |
| Burns : Electrical | Burns | Neurological : Stroke > 8 hours | Neurological |
| Burns : Inhalation | Burns | Neurological : Syncope | Neurological |
| Burns : Thermal | Burns | Neurological : Transient Ischemic Attack | Neurological |
| Cardiac Arrest | Cardiac arrest-Medical | Seizures | Neurological |
| Cardiac Problems | Cardiovascular | Non-specific problems | NonSpecificProblems |
| Cardiovascular : ACS Other | Cardiovascular | Nothing Abnormal Detected | NonSpecificProblems |
| Cardiovascular : ACS STEMI | Cardiovascular | Other : Nothing Abnormal Detected | NonSpecificProblems |
| Cardiovascular : Acute Pulmonary Edema | Cardiovascular | Non-trauma back pain | NonTraumaticBackPain |
| Cardiovascular : Angina Pectoris | Cardiovascular | Not Recorded | Not Recorded |
| Cardiovascular : Atrial Fibrillation | Cardiovascular | OBS GYN : Breech Presentation | OBS GYN |
| Cardiovascular : Congestive Heart Failure | Cardiovascular | OBS GYN : Delivery | OBS GYN |
| Cardiovascular : Hypertensive Emergency | Cardiovascular | OBS GYN : Eclampsia | OBS GYN |
| Cardiovascular : Narrow Complex Bradyarrhythmia | Cardiovascular | OBS GYN : Ectopic pregnancy | OBS GYN |
| Cardiovascular : Narrow Complex Tachyarrhythmia | Cardiovascular | OBS GYN : Hemorrhage | OBS GYN |
| Cardiovascular : Pericarditis | Cardiovascular | OBS GYN : Placenta Abruptio | OBS GYN |
| Cardiovascular : Wide Complex Bradyarrhythmia | Cardiovascular | OBS GYN : Placenta Previa | OBS GYN |
| Cardiovascular : Wide Complex Tachyarrhythmia | Cardiovascular | OBS GYN : Post Delivery Care | OBS GYN |
| Hypertensive Emergency | Cardiovascular | OBS GYN : Pre Term Labor | OBS GYN |
| Other : Exacerbation of Chronic Medical Condition | Chronic Medical Condition | OBS GYN : Pre-eclampsia | OBS GYN |
| Other : Medical Device Failure | Chronic Medical Condition | OBS GYN : Umbilical Cord Prolapse | OBS GYN |
| Other : Combative Patient | Combative Patient | Other : Pain Unknown Cause | Pain |
| Other : Confirmed COVID 19 | COVID19 related | Other : Pain, Unknown Cause | Pain |
| Other : Suspected COVID 19 | COVID19 related | Pain - non-trauma | Pain |
| Endocrine : Diabetic Ketoacidosis | Diabetic problem | Other: Parental concern | Parental concern |
| Endocrine : Hyperglycemia | Diabetic problem | Asthma | Respiratory |
| Endocrine : Hypoglycemia | Diabetic problem | Recreational drugs / Alcohol OD | Respiratory |
| Hypoglycemia | Diabetic problem | Respiratory : Asthma | Respiratory |
| D.O.A | DOA | Respiratory : Complete FBAO | Respiratory |
| Other : Medical Obvious Death/DOA | DOA | Respiratory : COPD | Respiratory |
| Trauma : Obvious Death/DOA | DOA | Respiratory : Croup | Respiratory |
| Other : Electrical Injury | Electrocution | Respiratory : Epiglottitis | Respiratory |
| Other : Hyperkalemia | Endocrinology | Respiratory : Hyperventilation Syndrome | Respiratory |
| Other : Nose Bleed | Epistaxix | Respiratory : Lower Respiratory Infection | Respiratory |
| Hyperthermia | Febrile Illness | Respiratory : Lower Respiratory Infrection | Respiratory |
| Other : Febrile Illlness | Febrile Illness | Respiratory : Partial FBAO | Respiratory |
| Abdominal pain | GIGU | Respiratory : Pleurisy | Respiratory |
| Abdominal problem | GIGU | Respiratory : Pneumothorax Medical | Respiratory |
| Genito -urinary problems | GIGU | Respiratory : Pulmonary Edema | Respiratory |
| GI GU : Acute Abdomen | GIGU | Respiratory : Upper AIrway Swelling | Respiratory |
| GI GU : Constipation | GIGU | Respiratory : Upper Respiratory Infection | Respiratory |
| GI GU : Gastroentiritis | GIGU | Shock : Distributive/Septic/Anaphylaxis | Shock |
| GI GU : Hematuria | GIGU | Shock : Hypovolemic | Shock |
| GI GU : Hernia | GIGU | Shock : Obstructive | Shock |
| GI GU : Liver Failure | GIGU | Dizziness | Sick person |
| GI GU : Renal Colic | GIGU | Headache | Sick person |
| GI GU : Upper GI Bleed | GIGU | Malaise | Sick person |
| GI GU : Urinary Tract Infection | GIGU | Medical Emergencies | Sick person |
| Hazmat : Absorption | HazMat | Envenomation | Toxicology |
| Hazmat : Ingestion | HazMat | Environmental : Envenomation | Toxicology |
| Hazmat : Inhalation | HazMat | Toxicological : Opioid | Toxicology |
| Hazmat : Radiation | HazMat | Toxicological : Organophosphate | Toxicology |
| Environmental : Heat Illness | Heat related | Toxicological : Other | Toxicology |
| Environmental : Hypothermia | Heat related | Toxicological : Sedative | Toxicology |
| Heat Stroke | Heat related | Toxicological : Unknown Substance | Toxicology |
| Non-Emergency Transport | IFT | Major Musculoskeletal Injury | Trauma |
| Other : Care by Medical Escort Team | IFT | Trauma - Thoracic | Trauma |
| Transport : IFT/PTS | IFT | Trauma : Abdominal | Trauma |
| Transport : Retrieval | IFT | Trauma : Amputation | Trauma |
| Other: Basic check-up | Minor illness | Trauma : Avulsion/Degloving | Trauma |
| Other: Minor illness (unspecified) | Minor illness | Trauma : Blast Injury | Trauma |
| Minor joint injury | Minor trauma | Trauma : Blunt Trauma | Trauma |
| Minor musculoskeletal injury | Minor trauma | Trauma : Brain Injury | Trauma |
| Minor Trauma | Minor trauma | Trauma : Closed Fracture | Trauma |
| Non-trauma musculoskeletal pain | Minor trauma | Trauma : Dislocation | Trauma |
| Other: Minor injury (unspecified) | Minor trauma | Trauma : Femur Fracture(s) | Trauma |
| Trauma - Soft Tissue Injury/Bruising/Abrasion | Minor trauma | Trauma : Head Injury | Trauma |
| Trauma - Sprain / Strain | Minor trauma | Trauma : Major Hemorrhage | Trauma |
| Trauma : Laceration | Minor trauma | Trauma : Pelvic fracture | Trauma |
| Trauma : Soft Tissue Injury/Bruising/Abrasion | Minor trauma | Trauma : Penetrating Trauma | Trauma |
| Trauma : Sprain / Strain | Minor trauma | Trauma : Spinal Injury | Trauma |
| Trauma pain | Minor trauma | Trauma : Thoracic | Trauma |
| Environmental : Submersion | Near Drowning | Trauma - Obvious Death/DOA | TraumaUndeniableDeath |
| Age categories | | | |
| 00-14/15-29/30-44/45-59/60-74/75-89/≥90 | | | |

# Annexe 2: Feature selection results using Chi_Saqure test

| Variables |  | Non-Transport Categories | | | Chi-Square (p-value) |
| --- | --- | --- | --- | --- | --- |
|  |  | 0  DOA | 1  (Refused transport and treatment) | 2  (Refused transport but treated on scene) |  |
| Kruskal Wallis | | | | | |
| Pearson's Chi-squared test with Yates' continuity correction | | | | | |
| Gender | 0 | 163(0.09) | 54527(28.68) | 19187(10.09) | 537.89 (2.2e-16) |
|  | 1 | 816(0.43) | 80887(42.55) | 34517(18.16) |  |
| Zone | 0 | 288(0.15) | 34377(18.08) | 13651(7.18) | 8.3325 (0.01551) |
|  | 1 | 691(0.36) | 101037(53.15) | 40053(21.07) |  |
| Priority | 0 | 908(0.48) | 97693(51.39) | 32400(17.04) | 2765.9 (2.2e-16) |
|  | 1 | 71(0.04) | 37721(19.84) | 21304(11.21) |  |
| <=14 | 0 | 933(0.49) | 119325(62.77) | 50070(26.34) | 1113.9 (2.2e-16) |
|  | 1 | 46(0.02) | 16089(8.46) | 3634(1.91) |  |
| >=90^*^ | 0 | 944(0.50) | 134806(70.91) | 53537(28.16) | 23.009 (1.613e-06) |
|  | 1 | 35(0.02) | 608(0.32) | 167(0.09) |  |
| 14-29] | 0 | 857(0.45) | 97516(51.30) | 36936(19.43) | 324.7 (2.2e-16) |
|  | 1 | 122(0.06) | 37898(19.94) | 16768(8.82) |  |
| ]29-44] | 0 | 592(0.31) | 88235(46.42) | 33340(17.54) | 164.85 (2.2e-16) |
|  | 1 | 387(0.20) | 47179(24.82) | 20364(10.71) |  |
| ]44-59] | 0 | 754(0.40) | 116861(61.47) | 45948(24.17) | 84.321 (2.2e-16) |
|  | 1 | 225(0.12) | 18553(9.76) | 7756(4.08) |  |
| ]59-74] | 0 | 858(0.45) | 125678(66.11) | 50334(26.48) | 94.043 (2.2e-16) |
|  | 1 | 121(0.06) | 9736(5.12) | 3370(1.77) |  |
| ]74-89] | 0 | 936(0.49) | 130063(68.42) | 52059(27.39) | 86.445 (2.2e-16) |
|  | 1 | 43(0.02) | 5351(2.81) | 1645(0.87) |  |
| 2018 | 0 | 870(0.46) | 123014(64.71) | 48932(25.74) | 8.3972 (0.01502) |
|  | 1 | 109(0.06) | 12400(6.52) | 4772(2.51) |  |
| 2019 | 0 | 799(0.42) | 110665(58.22) | 43668(22.97) | 4.3291 (0.1148) |
|  | 1 | 180(0.09) | 24749(13.02) | 10036(5.28) |  |
| 2020 | 0 | 770(0.41) | 99532(52.36) | 40176(21.13) | 45.639 (1.229e-10) |
|  | 1 | 209(0.11) | 35882(18.88) | 13528(7.12) |  |
| 2021 | 0 | 700(0.37) | 100094(52.65) | 38903(20.46) | 45.068(1.635e-10) |
|  | 1 | 279(0.15) | 35320(18.58) | 14801(7.79) |  |
| 2022 | 0 | 777(0.41) | 108351(57) | 43137(22.69) | 2.634(0.2679) |
|  | 1 | 202(0.11) | 27063(14.24) | 10567(5.56) |  |
| April | 0 | 837(0.44) | 115581(60.8) | 46154(24.28) | 10.724(0.00469) |
|  | 1 | 142(0.07) | 19833(10.43) | 7550(3.97) |  |
| August | 0 | 947(0.5) | 131592(69.22) | 52054(27.38) | 9.0319(0.01093) |
|  | 1 | 32(0.02) | 3822(2.01) | 1650(0.87) |  |
| December | 0 | 931(0.49) | 130262(68.52) | 51577(27.13) | 5.4442(0.06574) |
|  | 1 | 48(0.03) | 5152(2.71) | 2127(1.12) |  |
| February | 0 | 858(0.45) | 119571(62.9) | 47420(24.95) | 0.40995(0.8147) |
|  | 1 | 121(0.06) | 15843(8.33) | 6284(3.31) |  |
| January | 0 | 856(0.45) | 116174(61.11) | 46494(24.46) | 21.236 (2.447e-05) |
|  | 1 | 123(0.06) | 19240(10.12) | 7210(3.79) |  |
| July | 0 | 940(0.49) | 129675(68.22) | 51268(26.97) | 8.523(0.0141) |
|  | 1 | 39(0.02) | 5739(3.02) | 2436(1.28) |  |
| June | 0 | 846(0.45) | 118430(62.3) | 46704(24.57) | 9.125(0.01044) |
|  | 1 | 133(0.07) | 16984(8.93) | 7000(3.68) |  |
| March | 0 | 867(0.46) | 115786(60.91) | 46536(24.48) | 47.643(4.513e-11) |
|  | 1 | 112(0.06) | 19628(10.33) | 7168(3.77) |  |
| May | 0 | 858(0.45) | 117270(61.69) | 46151(24.28) | 15.75(0.0003801) |
|  | 1 | 121(0.06) | 18144(9.54) | 7553(3.97) |  |
| November | 0 | 953(0.5) | 131593(69.22) | 52047(27.38) | 9.7127(0.007779) |
|  | 1 | 26(0.01) | 3821(2.01) | 1657(0.87) |  |
| October | 0 | 939(0.49) | 131812(69.34) | 52244(27.48) | 7.9107(0.01915) |
|  | 1 | 40(0.02) | 3602(1.89) | 1460(0.77) |  |
| September | 0 | 931(0.49) | 130262(68.52) | 51577(27.13) | 24.375(5.093e-06) |
|  | 1 | 48(0.03) | 5152(2.71) | 2127(1.12) |  |
| East Asia & Pacific | 0 | 927(0.49) | 128232(67.46) | 51055(26.86) | 10.772 (0.004579) |
|  | 1 | 52(0.03) | 7182(3.78) | 2649(1.39) |  |
| Europe & Central Asia | 0 | 942(0.50) | 130403(68.60) | 51485(27.08) | 19.47 (5.917e-05 |
|  | 1 | 37(0.02) | 5011(2.64) | 2219(1.17) |  |
| Latin America and Caribbean* | 0 | 979(0.52) | 135158(71.10) | 53602(28.20) | 0.0018092 (0.9661) |
|  | 1 | 0(0) | 256(0.13) | 102(0.05) |  |
| MENA | 0 | 885(0.47) | 101547(53.42) | 41248(21.70) | 185.77 (2.2e-16) |
|  | 1 | 94(0.05) | 33867(17.82) | 12456(6.55) |  |
| North America | 0 | 959(0.5) | 133658(70.31) | 52909(27.83) | 13.229 (0.001341) |
|  | 1 | 20(0.01) | 1756(0.92) | 795(0.42) |  |
| Other* | 0 | 979(0.52) | 135376(71.21) | 53696(28.25) | 2.1679 (0.1409) |
|  | 1 | 0(0) | 38(0.02) | 8(0) |  |
| Other GCC | 0 | 946(0.5) | 130288(68.54) | 51792(27.25) | 5.7785 (0.05562) |
|  | 1 | 33(0.02) | 5126(2.7) | 1912(1.01) |  |
| Qatar | 0 | 858(0.45) | 101705(53.5) | 42471(22.34) | 406.29(2.2e-16) |
|  | 1 | 121(0.06) | 33709(17.73) | 11233(5.91) |  |
| South Asia | 0 | 486(0.26) | 97651(51.37) | 36164(19.02) | 631.95 (2.2e-16) |
|  | 1 | 493(0.26) | 37763(19.87) | 17540(9.23) |  |
| Sub Saharan Africa | 0 | 915(0.48) | 124792(65.65) | 48928(25.74) | 59.997 (9.373e-14) |
|  | 1 | 64(0.03) | 10622(5.59) | 4776(2.51) |  |
| Unknown^*^ | 0 | 914(0.48) | 135330(71.19) | 53690(28.24) | 30.151 (3.996e-08) |
|  | 1 | 65(0.03) | 84(0.04) | 14(0.01) |  |
| Abdominal Pain | 0 | 973(0.51) | 131093(68.96) | 49454(26.02) | 2025.5 (2.2e-16) |
|  | 1 | 6(0) | 4321(2.27) | 4250(2.24) |  |
| Abnormal Behaviour | 0 | 972(0.51) | 133582(70.27) | 53457(28.12) | 283.85(2.2e-16) |
|  | 1 | 7(0) | 1832(0.96) | 247(0.13) |  |
| Allergic Reaction | 0 | 978(0.51) | 134202(70.60) | 53336(28.06) | 26.871(1.462e-06) |
|  | 1 | 1(0) | 1212(0.64) | 368(0.19) |  |
| Animal Attack Bite* | 0 | 979(0.52) | 135295(71.17) | 53651(28.22) | 0.43858(0.5078) |
|  | 1 | 0(0) | 119(0.06) | 53(0.03) |  |
| Assault | 0 | 976(0.51) | 131902(69.39) | 52339(27.53) | 20.52 (3.501e-05) |
|  | 1 | 3(0) | 3512(1.85) | 1365(0.72) |  |
| Back Pain | 0 | 972(0.51) | 133216(70.08) | 50846(26.75) | 2038.5(2.2e-16) |
|  | 1 | 7(0) | 2198(1.16) | 2855(1.50) |  |
| Bleeding | 0 | 979(0.52) | 133952(70.47) | 53140(29.95) | 10.917(0.004259) |
|  | 1 | 0(0) | 1462(0.77) | 564(0.30) |  |
| Breathing Problem | 0 | 977(0.51) | 124943(65.73) | 27.95(26.45) | 178.11(2.2e-16) |
|  | 1 | 2(0) | 10471(5.51) | 3423(1.80) |  |
| Cardiac Arrest | 0 | 357(0.19) | 134996(71.01) | 53608(28.20) | 65630(2.2e-16) |
|  | 1 | 622(0.33) | 418(0.22) | 96(0.05) |  |
| Chest Pain | 0 | 974(0.51) | 126685(66.64) | 50641(26.64) | 91.272(2.2e-16) |
|  | 1 | 5(0) | 8729(4.59) | 3063(1.61) |  |
| Chocking | 0 | 964(0.51) | 129404(68.07) | 52214(27.47) | 295.53(2.2e-16) |
|  | 1 | 15(0.01) | 6010(3.16) | 1490(0.78) |  |
| Diabetic Problem | 0 | 979(0.52) | 133156(70.05) | 52897(27.83) | 22.71(1.171e-05) |
|  | 1 | 0(0) | 2258(1.19) | 807(0.42) |  |
| Drowning Near Drowning^*^ | 0 | 979(0.52) | 135305(71.18) | 53699(28.25) | 30.883 (2.74e-08) |
|  | 1 | 0(0) | 109(0.06) | 5(0) |  |
| Electrocution Lightening^*^ | 0 | 974(0.51) | 134848(70.94) | 53626(28.21) | 83.852 (2.2e-16) |
|  | 1 | 5(0) | 566(0.30) | 78(0.04) |  |
| Entrapment* | 0 | 972(0.51) | 134870(70.95) | 53653(28.22) | 115.58(2.2e-16) |
|  | 1 | 7(0) | 544(0.29) | 51(0.03) |  |
| Eye Problem* | 0 | 979(0.52) | 135007(71.02) | 53585(28.19) | 7.9641 (0.004771) |
|  | 1 | 0(0) | 407(0.21) | 119(0.06) |  |
| Fall | 0 | 947(0.5) | 131159(69) | 51767(27.23) | 26.209(2.036e-06) |
|  | 1 | 32(0.02) | 4255(2.24) | 1937(1.02) |  |
| Fire Burn | 0 | 979(0.52) | 133804(70.39) | 52918(27.84) | 35.866(1.629e-08) |
|  | 1 | 0(0) | 1610(0.85) | 786(0.41) |  |
| HazMat^*^ | 0 | 979(0.52) | 134765(70.89) | 53525(28.16) | 17.721(2.558e-05) |
|  | 1 | 0(0) | 649(0.34) | 179(0.09) |  |
| Headache | 0 | 977(0.51) | 133204(70.07) | 52038(27.37) | 432.49(2.2e-16) |
|  | 1 | 2(0) | 2210(1.16) | 1666(0.88) |  |
| Heart Problems | 0 | 977(0.51) | 131773(69.32) | 52950(27.85) | 300.29(2.2e-16) |
|  | 1 | 2(0) | 3641(1.92) | 754(0.40) |  |
| Heat-Related | 0 | 974(0.51) | 134767(70.89) | 53346(28.06) | 25.951(2.317e-06) |
|  | 1 | 5(0) | 647(0.34) | 358(0.19) |  |
| IFT Health Center^*^ | 0 | 966(0.51) | 134707(70.86) | 53603(28.20) | 102.68(2.2e-16) |
|  | 1 | 13(0.01) | 707(0.37) | 101(0.05) |  |
| IFT-Hospital** | 0 | 977(0.51) | 135411(71.23) | 53701(28.25) | 0.6951 |
|  | 1 | 3(0) | 3(0) | 3(0) |  |
| Pandemic | 0 | 958(0.5) | 110875(58.33) | 46766(24.60) | 901 (2.2e-16) |
|  | 1 | 21(0.01) | 24539(12.91) | 6938(3.65) |  |
| Poisoning^*^ | 0 | 973(0.51) | 134984(71.01) | 53578(28.18) | 141(2.2e-16) |
|  | 1 | 6(0) | 430(0.23) | 126(0.07) |  |
| Pregnancy* | 0 | 973(0.51) | 134984(71.01) | 53578(28.18) | 9.1684(0.002462) |
|  | 1 | 6(0) | 430(0.23) | 126(0.07) |  |
| RTA | 0 | 961(0.51) | 121678(64.01) | 49341(25.96) | 249.42(2.2e-16) |
|  | 1 | 18(0.01) | 13736(7.23) | 4363(2.30) |  |
| Seizure | 0 | 889(0.47) | 131088(68.96) | 52653(27.70) | 351.46(2.2e-16) |
|  | 1 | 90(0.05) | 4326(2.28) | 1051(0.55) |  |
| Sick Person | 0 | 953(0.5) | 121147(63.73) | 46339(24.38) | 459.18(2.2e-16) |
|  | 1 | 26(0.01) | 14267(7.51) | 7365(3.87) |  |
| Stabbing Gunshot^*^ | 0 | 969(0.51) | 135367(71.21) | 53687(28.24) | 0.77354(0.3791) |
|  | 1 | 10(0.01) | 47(0.02) | 17(0.01) |  |
| Strock^*^ | 0 | 979(0.52) | 134859(70.94) | 53591(28.19) | 41.93(9.462e-11) |
|  | 1 | 0(0) | 555(0.29) | 113(0.06) |  |
| Traumatic injury | 0 | 972(0.51) | 132368(69.63) | 51438(27.06) | 564.42(2.2e-16) |
|  | 1 | 7(0) | 3046(1.6) | 2266(1.19) |  |
| Unconscious | 0 | 958(0.5) | 134882(70.95) | 53556(28.17) | 426.79(2.2e-16) |
|  | 1 | 21(0.01) | 532(0.28) | 148(0.08) |  |
| Unknown Problem^*^ | 0 | 958(0.50) | 134882(70.95) | 53556(28.17) | 17.335(3.134e-05) |
|  | 1 | 21(0.01) | 532(0.28) | 148(0.08) |  |
| Walking Patient | 0 | 968(0.51) | 129151(67.94) | 49371(25.97) | 902.43(2.2e-16) |
|  | 1 | 11(0.01) | 6263(3.29) | 4333(2.28) |  |
| Alpha | 0 | 62(0.03) | 15440(8.12) | 9624(5.06) | 1465.1(2.2e-16) |
|  | 1 | 917(0.48) | 119974(63.11) | 44080(23.19) |  |
| Bravo | 0 | 977(0.51) | 127701(67.18) | 45776(24.08) | 4269.6 (2.2e-16) |
|  | 1 | 2(0) | 7713(4.06) | 7928(4.17) |  |
| COVID* | 0 | 979(0.52) | 135363(71.21) | 53671(28.23) | 4.5185(0.03353) |
|  | 1 | 0(0) | 51(0.03) | 33(0.02) |  |
| Charlie^*^ | 0 | 970(0.51) | 135229(71.14) | 53636(28.22) | 0.57397(0.4487) |
|  | 1 | 9(0) | 185(0.10) | 68(0.04) |  |
| Delta^*^ | 0 | 977(0.51) | 135075(71.06) | 53649(28.22) | 39.674(3e-10) |
|  | 1 | 2(0) | 339(0.18) | 55(0.03) |  |
| Event | 0 | 979(0.52) | 134264(70.63) | 53120(27.94) | 33.197(6.184e-08) |
|  | 1 | 0(0) | 1150(0.60) | 584(0.31) |  |
| Foxtrot^*^ | 0 | 967(0.51) | 135192(71.12) | 53647(28.22) | 10.367(0.001283) |
|  | 1 | 12(0.01) | 222(0.12) | 57(0.03) |  |
| Green bus^*^ | 0 | 978(0.51) | 134633(70.82) | 53650(28.22) | 195.64(2.2e-16) |
|  | 1 | 1(0) | 781(0.41) | 54(0.03) |  |
| SEM | 0 | 945(0.5) | 130504(68.65) | 52870(27.81) | 561.32(2.2e-16) |
|  | 1 | 34(0.02) | 4910(2.58) | 834(0.44) |  |
| Tango^*^ | 0 | 977(0.51) | 135325(71.19) | 53693(28.25) | 14.51(0.0001394) |
|  | 1 | 2(0.00) | 89(0.05) | 11(0.01) |  |
| Allergic Reaction | 0 | 978(0.51) | 134313(70.65) | 53417(28.10) | 46.524(7.898e-11) |
|  | 1 | 1(0) | 1101(0.58) | 287(0.15) |  |
| Anaphylaxis* | 0 | 979(0.52) | 135369(71.21) | 53669(28.23) | 8.7355(0.003121) |
|  | 1 | 0(0) | 45(0.02) | 35(0.02) |  |
| Animal Bite | 0 | 977(0.51) | 134087(70.54) | 52887(27.82) | 108.5(2.2e-16) |
|  | 1 | 2(0) | 1327(0.7) | 817(0.43) |  |
| Burns | 0 | 974(0.51) | 12883(67.77) | 52373(27.55) | 577.95(2.2e-16) |
|  | 1 | 5(0) | 6576(3.46) | 1331(0.70) |  |
| COVID19 related^*^ | 0 | 782(0.41) | 135391(71.22) | 53687(28.24) | 50.975(9.355e-13) |
|  | 1 | 197(0.1) | 23(0.01) | 17(0.01) |  |
| Cardiac Arrest Medical | 0 | 972(0.51) | 132976(69.95) | 53048(27.91) | 85.455(2.2e-16) |
|  | 1 | 7(0) | 2438(1.28) | 656(0.35) |  |
| Cardiovascular | 0 | 975(0.51) | 133038(69.98) | 52888(27.82) | 22.509(1.295e-05) |
|  | 1 | 4(0) | 2376(1.25) | 816(0.43) |  |
| Chronic Medical Condition* | 0 | 979(0.52) | 135381(71.22) | 53698(28.25) | 2.5825(0.1081) |
|  | 1 | 0(0) | 33(0.02) | 6(0.00) |  |
| Combative patient^*^ | 0 | 324(0.17) | 135370(71.21) | 53704(28.25) | 274.85(2.2e-16) |
|  | 1 | 655(0.34) | 44(0.02) | 0(0) |  |
| DOA | 0 | 979(0.52) | 133254(70.10) | 52591(27.67) | 69.025(1.026e-15) |
|  | 1 | 0(0) | 2160(1.14) | 1113(0.59) |  |
| Diabetic Problem* | 0 | 979(0.52) | 135365(71.21) | 53690(28.24) | 0.85205(0.356) |
|  | 1 | 0(0) | 49(0.03) | 14(0.01) |  |
| Electrocution** | 0 | 979(0.52) | 135408(71.23) | 53704(28.25) | 0.1939 |
|  | 1 | 0(0) | 6(0) | 0(0) |  |
| Endocrinology | 0 | 979(0.52) | 134723(70.87) | 53402(28.09) | 7.1726(0.0277) |
|  | 1 | 0(0) | 691(0.36) | 302(0.16) |  |
| Epistaxix | 0 | 970(0.51) | 130145(68.46) | 50869(26.76) | 7.1726(0.0277) |
|  | 1 | 9(0) | 5269(2.77) | 2835(1.49) |  |
| Febrile Illiness | 0 | 961(0.51) | 127489(67.07) | 47406(24.94) | 208.3(2.2e-16) |
|  | 1 | 18(0.01) | 7925(4.17) | 6298(3.31) |  |
| GIGU^*^ | 0 | 979(0.52) | 134745(70.88) | 53502(28.14) | 10.799 (0.001015) |
|  | 1 | 0(0) | 669(0.35) | 202(0.11) |  |
| HazMat^*^ | 0 | 977(0.51) | 134952(70.99) | 53323(28.05) | 117.89(2.2e-16) |
|  | 1 | 2(0) | 462(0.24) | 381(0.2) |  |
| Heat Related* | 0 | 977(0.51) | 135341(71.2) | 53696(28.25) | 13.287(0.0002672) |
|  | 1 | 2(0) | 73(0.04) | 8(0) |  |
| IFT | 0 | 978(0.51) | 126029(66.3) | 52353(27.54) | 1463.3(2.2e-16) |
|  | 1 | 1(0) | 9385(4.94) | 1351(0.71) |  |
| Minor Illiness | 0 | 964(0.51) | 118316(62.24) | 43422(22.84) | 1457.3(2.2e-16) |
|  | 1 | 15(0.01) | 17098(8.99) | 10282(5.41) |  |
| Minor Trauma** | 0 | 979(0.52) | 135403(71.23) | 53704(28.25) | 0.04132 |
|  | 1 | 0(0) | 11(0.01) | 0(0) |  |
| Near Drowning | 0 | 968(0.51) | 125902(66.23) | 48705(25.62) | 343.53(2.2e-16) |
|  | 1 | 11(0.01) | 9512(5) | 4999(2.63) |  |
| Neurological | 0 | 970(0.51) | 98253(51.69) | 49293(25.93) | 8573(2.2e-16) |
|  | 1 | 9(0) | 37161(19.55) | 4411(2.32) |  |
| Non specific problems** | 0 | 979(0.52) | 135413(71.23) | 53702(28.25) | 0.1943 |
|  | 1 | 0 | 1(0) | 2(0) |  |
| Non traumatic Back Pain* | 0 | 979(0.52) | 135404(10) | 53694(28.25) | 3.6564 (0.05585) |
|  | 1 | 0(0) | 10(0.01) | 10(0.01) |  |
| Not recorded* | 0 | 979(0.52) | 135194(71.12) | 53662(28.23) | 18.73(1.506e-05) |
|  | 1 | 0(0) | 220(0.12) | 42(0.02) |  |
| OBS GYN | 0 | 966(0.51) | 118332(62.25) | 42058(22.12) | 2611.3(2.2e-16) |
|  | 1 | 13(0.01) | 17082(8.99) | 11646(6.13) |  |
| Pain^*^ | 0 | 979(0.52) | 135272(71.16) | 53688(28.24) | 24.739(6.563e-07) |
|  | 1 | 0(0) | 142(0.07) | 16(0.01) |  |
| Parental concern | 0 | 970(0.51) | 128191(67.43) | 50579(26.61) | 56.736(4.786e-13) |
|  | 1 | 9(0) | 7223(3.80) | 3125(1.64) |  |
| Respiratory^*^ | 0 | 978(0.51) | 135383(71.22) | 53662(28.23) | 28.287(1.046e-07) |
|  | 1 | 1(0) | 31(0.02) | 42(0.02) |  |
| Shock* | 0 | 979(0.52) | 135373(71.21) | 53683(28.24) | 0.70899 (0.3998) |
|  | 1 | 0(0) | 41(0.02) | 21(0.01) |  |
| Sick Person^*^ | 0 | 977(0.51) | 134761(70.89) | 53619(28.21) | 102.17(2.2e-16) |
|  | 1 | 2(0) | 653(0.34) | 85(0.04) |  |
| Toxicology | 0 | 963(0.51) | 129837(68.30) | 51150(26.91) | 54.928(1.182e-12) |
|  | 1 | 16(0.01) | 5577(2.93) | 2554(1.34) |  |
| *With 2by2 matrix **Fisher’s Exact Test | | | | | |

Annexe 3: Missing data assement results

|  | MissingValue (%) | | | | | | | | | |
| --- | --- | --- | --- | --- | --- | --- | --- | --- | --- | --- |
|  | 2018 | 2019 | | | 2019 | 2020 | | 2021 | | 2022 |
| Expr1001 |  | | | 100% | | | | | | |
| CFS_ID | 0% | | | | | | | | | |
| Dispatch_Code |  |  |  |  |  |  |  |  |  |  |
| Creation_DT |  |  |  |  |  |  |  |  |  |  |
| Pending_Dispatch_DT |  |  |  |  |  |  |  |  |  |  |
| Active_Dispatch_DT |  |  |  |  |  |  |  |  |  |  |
| En_Route_DT | 13958(27.738%) | 12675(25.719%) | 1340(24.693%) | | | | 13512(20.618%) | | 9850(15.030%) | 9297(18.845%) |
| WithPatient_DT | 34951(69.456%) | 33512(68%) | 34734(63.865%) | | | | 38153(58.219%) | | 30395(46.381%) | 22968(46.556%) |
| ToDestination_P1_DT | 50210(99.779%) | (49199(99.832%) | 54233(99.717%) | | | | 65429(99.840%) | | 65337(99.699%) | 49229(99.787%) |
| At_Dest_DT | 47985(95.359%) | 47269(95.915%) | 51961(95.539%) | | | | 63221(96.471%) | | 63131(96.333%) | 47199(95.672%) |
| Assigned_Available_DT | 1704(3.386%) | 2464(5%) | 2676(4.920%) | | | | 2105(3.212%) | | 1461(2.229%) | 844(1.711%) |
| Rejected_DT | 50321(100%) | 49282(100%) | 54387(100%) | | | | 65534 (100%) | | 65534(100%) | 49334(100%) |
| 1st_AS_DT | 0% | 0% | 0% | | | | 0% | | 0% | 0% |
| Zone_T | 50321(100%) | 49282(100%) | 54387(100%) | | | | 65534 (100%) | | 65534(100%) | 49334(100%) |
| Zone_No | 0% | | | | | | | | | |
| Priority_ |  |  |  |  |  |  |  |  |  |  |
| Date |  |  |  |  |  |  |  |  |  |  |
| PatientID |  |  |  |  |  |  |  |  |  |  |
| PCRTime | 98(0.195%) | 104(0.211%) | 127(0.234%) | | | | 63(0.096%) | | 33(0.050%) | 6(0.012%) |
| PriorityToScene | 963(1.914%) | 1028(2.086%) | 999(1.837%) | | | | 932(1.422%) | | 731(1.115%) | 600(1.216%) |
| PriorityToHospital | 45753(90.922%) | 45466(92.257%) | 49935(91.814%) | | | | 59881(91.374%) | | 61564(93.942%) | 45833(92.903%) |
| Gender | 20(0.040%) | 10(0.020%) | 11(0.020%) | | | | 8(0.012%) | | 5(0.008%) | 11(0.022%) |
| Nationality | 27142(53.938%) | 28124(57.067%) | 28434(52.281%) | | | | 1224(1.868%) | | 1096(1.672%) | 978(1.982%) |
| Age | 6(0.012%) | 1(0.002%) | 8(0.015%) | | | | 0% | | 0% | 0% |
| ProvisionalDiagnosis | 2246(4.463%) | 1705(3.460%) | 1209(2.223%) | | | | 336(0.513%) | | 26(0.040%) | 22(0.045%) |
| Handover | 0% | 14(0.028%) | 0% | | | | 0% | | 0% | 0% |
| Group |  | 48285(97.977%) | 53461(98.297%) | | | | 64534(98.474%) | | 64474(98.383%) | 48324(97.953%) |
| TansportUnit | 0% | | | | | | | | | |
| Year |  |  |  |  |  |  |  |  |  |  |
